# Characterization of the SIM-A9 cell line as a model of activated microglia in the context of neuropathic pain

**DOI:** 10.1371/journal.pone.0231597

**Published:** 2020-04-14

**Authors:** Kandarp M. Dave, Lalah Ali, Devika S. Manickam

**Affiliations:** Graduate School of Pharmaceutical Sciences, School of Pharmacy, Duquesne University, Pittsburgh, PA, United States of America; Università di Napoli Federico II, ITALY

## Abstract

Resident microglia of the central nervous system are being increasingly recognized as key players in diseases such as neuropathic pain. Biochemical and behavioral studies in neuropathic pain rodent models have documented compelling evidence of the critical role of ATP mediated-P2X4R-brain-derived neurotrophic factor (BDNF) signaling pathway in the initiation and maintenance of pain hypersensitivity, a feature driving neuropathic pain-related behavior. The goal of this study was to develop and characterize an *in vitro* cell line model of activated microglia that can be subsequently utilized for screening neuropathic pain therapeutics. In the present study, we characterized the SIM-A9 microglia cell line for key molecules in the P2X4R-BDNF signaling axis using a combination of biochemical techniques and developed an ATP-activated SIM-A9 microglia model. We present three novel findings: *first*, SIM-A9 cells expressed P2X4R and BDNF proteins, *second*, ATP, but not LPS, was cytocompatible with SIM-A9 cells and *third*, exposure of cells to optimized ATP concentrations for defined periods increased intracellular expression of Iba1 and BDNF proteins. Increased Iba1 levels confirmed microglia activation and increased BDNF expression confirmed ATP-mediated stimulation of the P2X4R signaling pathway. We propose that this ATP-activated SIM-A9 cell line model system can be utilized for screening both small- as well as macro-molecular neuropathic pain therapeutics targeting BDNF and/or P2X4R knockdown.

## Introduction

Microglia, the resident macrophages and innate immune cells of the central nervous system (CNS), are gaining increasing attention for their essential roles in adult brain development, maturation, and CNS disorders [1–3]. Microglia account for approximately 10% of total brain cells and play fundamental roles during various processes such as neuronal development, adult neurogenesis, modulating synaptic transmission, neuroprotection and neuroimmune interactions [2, 4]. Under physiological conditions, resting microglia exhibit a ramified small cellular morphology and remove damaged or unnecessary neurons and synapses, and continuously scan the CNS microenvironment without disturbing the neuronal synapses [2, 5]. Microglial morphology is readily responsive to any threatening events to the CNS homeostasis, such as peripheral nerve injury, trauma, or the presence of pathogens [2]. As a consequence, microglia undergo morphological, genetic, and functional changes exhibiting migratory, proliferative and phagocytic properties along with the release of neurotrophic factors, chemo or cytokines, and receptor stimulation—a state referred to as “activated” microglia [2, 5, 6]. A large body of published research has documented the role of activated microglia in the pathophysiology of neurodegenerative disorders, such as Alzheimer's disease [1], Huntington’s disease [4], Parkinson's disease [7–9], amyotrophic lateral sclerosis [4], neuroinflammation [10, 11], and also neuropathic pain [12–14].

Neuropathic pain characterized by tactile allodynia and hyperalgesia is a severely debilitating type of chronic pain that develops after persistent peripheral nerve damage [15, 16]. Patients with neuropathic pain often develop psychological consequences such as major depression, anxiety, sleep disturbance along with physical and emotional breakdown resulting in a poor quality of life [17]. Even first-line therapies such as gabapentin do not effectively treat the disease and the severe side effects make neuropathic pain notoriously difficult to treat amongst the other types of chronic pain [18]. Safe and specific therapies targeting the underlying pathogenesis of neuropathic pain is an urgent yet unmet need.

Here, we briefly present the central role of microglia in chronic neuropathic pain. Biochemical and behavioral studies in neuropathic pain rodent models provide compelling evidence that brain-derived neurotrophic factor (BDNF) released from microglia is a critical signaling molecule in microglia-neuron interaction mediating the pathogenesis of neuropathic pain caused by peripheral nerve injury (PNI) [12–14, 16, 19]. PNI activates microglia in the spinal dorsal horn marked by the upregulation of P2X4 purinoceptors (P2X4R). P2X4R is a subtype of ligand-gated ionotropic ATP receptors which when activated by extracellular ATP results in non-selective cation (Ca^2+^, Na^+^, and K^+^) flux across the cation channels [20, 21]. Activation of P2X4R increases the synthesis and release of BDNF from microglia that stimulates the nociceptive neurons in lamina I region of the spinal dorsal horn. The processes lead to the upregulation of downstream pathways in the CNS that produce pain hypersensitivity, a hallmark feature of neuropathic pain [12–14, 21]. Therefore, we propose that knockdown of BDNF expression in microglia using siRNA (siBDNF) can be a promising strategy for treating neuropathic pain. Our laboratory is developing lipidoid nanoparticles (LNP [22]) for the delivery of siBDNF to activated microglia as a safe and novel therapy for neuropathic pain. Lipid nanoparticles containing ionizable amino lipids are the delivery vehicle used in the first clinically available siRNA drug, ONPATTRO™, that was recently approved by the FDA for the treatment of amyloidosis [23, 24]. Noteworthy, LNPs have previously demonstrated safety and delivery efficacy both in *in vitro* and *in vivo* studies [22].

A robust and reliable *in vitro* model system mimicking activated microglial phenotypes will enable high-throughput screening of LNP/siBDNF formulations for treating neuropathic pain. Though reliable, *in vitro* studies utilizing primary microglia has significant challenges associated with time-consuming and expensive isolation methods, inadequate yield, purity, and intra-animal variability [1, 4]. On the other hand, many models of microglia-like cell lines have been used to characterize activation-mediated synthesis and secretion of biomolecules associated with different neurodegenerative disorders [1, 4, 25]. The most widely used microglia cell lines include the retrovirally transformed immortalized microglia BV-2 and N9 derived from rat and mouse [1], respectively. Li *et al*. developed an *in vitro* microglia neuroinflammation model using BV-2 and N9 cell lines [26]. BV-2 and N9 cells were exposed to lipopolysaccharide (LPS, 100 ng/mL) for different times that resulted in microglial activation showing increased expression of the microglial activation marker, ionized calcium-binding adapter molecule (Iba1) as well as increased expression of pro-nerve growth factor, and pro-brain derived neurotrophic factor (proBDNF). While they serve as useful models, virally transformed cell lines might alter the microglial phenotype and thus possess only short-term microglial properties [27].

Nagamoto-Combs *et al*. developed a novel, spontaneously Immortalized Microglia-A9 cell line (SIM-A9) from a primary glial culture of postnatal murine cerebral cortices [27]. Genetically or pharmacologically non-transformed SIM-A9 cells exhibited microglial phenotypes, including markers such as Iba1 and CD68, and retained microglial characteristics for as long as 40 passages. Also, LPS-stimulated SIM-A9 showed increased secretion of inflammatory mediators, such as tumor necrosis factor-α along with phagocytic activity similar to cultured primary microglia [27]. LPS or interleukin (IL)-1β-stimulated SIM-A9 cell line was utilized in inflammatory *in vitro* studies to analyze the changes in intracellular and/or secretory inflammatory mediators such as Tumor Necrosis Factor-α, cyclooxygenase-2, IL-6, NF-κB, various chemokines, and cytokines [28–30]. Since the SIM-A9 cell line exhibits key characteristics of primary microglia, we wanted to study its capability to simulate activated microglia in the context of the ATP-P2X4R-BDNF signaling axis in the context of peripheral nerve injury (PNI). PNI upregulates vesicular nucleotide transporters (VNUT) in spinal dorsal horn neurons which release ATP into the extracellular matrix within the spinal cord [21]. The VNUT-dependent extracellular ATP binds to microglial P2X4Rs leading to pain hypersensitivity and tactile allodynia [21]. Trang *et al*. reported that ATP-mediated stimulation of microglial P2X4Rs increased synthesis and calcium-dependent exocytic release of BDNF in primary microglia [31].

Our goal is to characterize the SIM-A9 cell line in the context of the P2X4R-BDNF signaling axis. In the present study, we characterized and validated the expression of the microglial activation marker Iba1, P2X4R—the purinergic receptor involved in ATP-mediated stimulation and the subsequent expression of BDNF in SIM-A9 microglia in resting and activated states. We used a combination of techniques such as immunocytochemistry (ICC), western blotting, and in-cell western (ICW) to characterize the above proteins in SIM-A9 cells. LPS and ATP were tested as stimulants to activate P2X4R. Cytocompatibility of a wide range of LPS and ATP concentrations were examined using luminescence-based ATP and absorbance-based MTS assays. Finally, the effect of ATP concentration on the activation of P2X4R and the subsequent expression of Iba1 and BDNF was evaluated using ICC, western blot, and ICW techniques. In summary, we have characterized a new cell line, SIM-A9, as an *in vitro* model of activated microglia in the context of the P2X4R-BDNF signaling pathway involved in PNI-mediated chronic neuropathic pain.

## Materials and methods

### Chemicals and bioreagents

Adenosine triphosphate (ATP) and paraformaldehyde were purchased from MP Biomedicals (Illkirch, France). Odyssey blocking buffer was procured from LI-COR (Lincoln, NE). Triton X-100, Tween-20, Ammonium Persulfate (APS), Tris hydrochloride, Tris base and sodium dodecyl sulfate (SDS) were purchased from Fisher Scientific (Pittsburgh, PA). Acrylamide 30% (Protogel) was purchased from National Diagnostics (Atlanta, GA) and N, N, N’, N’-Tetramethylethylenediamine was purchased from ACROS Organics (Ward Hill, MA). Nitrocellulose blocking membrane (Amersham Protran, 0.45 μm) was obtained from GE Healthcare Life Sciences, Germany. Aprotinin solution (10000 KIU/mL) was received from Fisher Bioreagents, New Zealand. RIPA buffer, 5x, was purchased from Alfa Aesar (Ward Hill, MA). Pierce BCA protein assay kit for total protein measurement was purchased from Thermo Scientific (Rockford, IL). The SDS-polyacrylamide gel was prepared using Mini-PROTEAN 1.5mm glass plates and Precision Plus protein standards were purchased from Bio-Rad (Hercules, CA) and 4X Laemmli SDS reducing sample buffer was received from Alfa Aesar (Ward Hill, MA). Mouse polyclonal to P2X4R (catalog (cat.) #ab168939) from Abcam, rabbit polyclonal to BDNF (cat.# PA5-15198) from Thermo scientific, rabbit polyclonal to Iba1 (cat.# 019–19741) from WAKO, and mouse monoclonal anti-alpha tubulin antibody (cat.# ab7291) from Abcam were used as primary antibodies. Hoechst 33342 (cat.# 62249) and DRAQ5™ (cat.# 62251) were purchased from Thermo Scientific (Germany and Rockford, IL respectively). Donkey anti-mouse Alexa Fluor (AF) 790 (cat.#715-655-150), AF680 (cat.#715-625-150), goat anti-rabbit AF790 (cat.#111-655-144) were purchased from Jackson ImmunoResearch Lab Inc. (West Grove, PA). Goat anti-rabbit IgG AF 555 (cat.#A21429) and goat anti-mouse IgG AF488 (cat.#A11001) were purchased from Life Technologies (Eugene, OR). Human BDNF protein (cat.#C076) was purchased from Novoprotein (Summit, NJ). Branched polyethyleneimine (PEI, molecular weight 25,000 Da) was purchased from Sigma-Aldrich, (St. Louis, MO). ATP assay was performed using a Cell Titer Glo 2.0 reagent (cat.#G9242, Promega, Madison, WI). Phenazine methosulfate (PMS) was obtained from Sigma-Aldrich (St. Louis, MO) and Cell Titer MTS [3-(4,5-dimethylthiazol-2-yl)-5-(3-carboxymethoxyphenyl)-2-(4-sulfophenyl)-2H-tetrazolium] reagent was obtained from Promega (Madison, WI). Methanol and 2-propanol were purchased from Fisher Scientific (Pittsburgh, PA). All the chemicals used for the experiments were molecular biology grade, DNase, RNase, and protease-free materials.

### Cell lines and cell culture

Spontaneously immortalized microglia, SIM-A9 cell line (cat.#CVCL_5I31) was purchased from Kerafast (Boston, MA) and U-87 MG (ATCC HTB-14) cell line was purchased from ATCC (Manassas, VA). SIM-A9 cells were maintained in complete growth media containing Dulbecco’s Modified Eagle Medium/F12 (DMEM/F12, HyClone, Logan, UT) supplemented with L-glutamine, sodium pyruvate, heat-inactivated fetal bovine serum; 10% (FBS, HyClone) and horse serum; 5% (HS, Gibco Molecular Probes, New Zealand). The media were protected against microbial contamination using 1.5 μg/mL penicillin and 1.5 U/mL streptomycin (Pen Strep, Gibco Life Technologies Corp., Grand Island, NY). The cells were incubated in a humidified 5% CO_2_ incubator at 37±0.5°C (Isotemp, Thermo Fisher Scientific). Prior to passaging, the cells were washed using 1x Modified Dulbecco’s Phosphate Buffered Saline; DPBS (HyClone) and were dissociated using 1x enzyme-free Cell Dissociation Buffer containing 1mM EGTA (EMD Millipore Corp, MA), 1mM EDTA (Fisher Bioreagents, Darmstadt, Germany) and 1mg/mL dextrose in DPBS.

### Immunocytochemistry (ICC) for P2X4R, Iba1, and BDNF protein detection

SIM-A9 cells were seeded at a density of 16,500 cells/200 μL/well in complete growth media (DMEM/F12 GlutaMAX +10%FBS+5%HS) in 96-well plates and were cultured for 48 h. The plate was transferred to a fume hood prior to fixation. In each well, 200 μL media was replaced with 200 μL 4% paraformaldehyde (PFA) solution (PFA was dissolved in DPBS at 70°C for 3 h on a hot-plate magnetic stirrer) and the cells were fixed for 20–25 min. The PFA solution was discarded and the fixed cells were washed thrice with 200 μL 10 mM PBS pH 7.4 for 10 min each. In each well, the cells were blocked with 50 μL blocking solution (Odyssey blocking buffer: 10 mM PBS pH 7.4::1:1) containing 0.3% v/v Triton X-100 for 1 h. The blocking solution was discarded from all the wells except for the secondary antibody-alone control groups (where primary antibodies were not added). Primary antibodies at 1:500 dilution in blocking solution were added in a volume of 50 μL/well and the plate was incubated at 2–8°C overnight without shaking. On the next day, the primary antibody and blocking solutions were discarded, and wells were washed thrice with 200 μL 10mM PBS pH 7.4 for 10 min each. A 50 μL/well mixture of secondary antibodies (BDNF and Iba1: goat anti-rabbit AF555; P2X4R: goat anti-mouse AF488) at 1:700 dilution in a blocking solution containing Hoechst (20 mM) at a 1:3000 dilution was added to each well and covered with aluminum foil to protect from light. The plate was incubated at room temperature (RT) for an hour. The secondary antibody solution was discarded, and cells were washed thrice with 200 μL 10 mM PBS pH 7.4 for 10 min each. The plate was imaged under phase contrast settings, DAPI (excitation 357/44 nm and emission 447/60 nm) for nucleus staining, and Green/Red Fluorescence channels (GFP, excitation 470/22 nm and emission 510/42 nm; RFP, excitation 531/40 nm and emission 593/40 nm) for target protein detection using an EVOS FL microscope (Life Technologies, Bothell, WA).

### Western blotting for characterization of P2X4R, Iba1, and BDNF proteins

#### 1. Cell lysate preparation

SIM-A9 cells were seeded in complete growth media at a density of 100,000 cells per mL per well in a 24-well plate and were cultured in a humidified incubator (37°C + 5% CO_2_) for 48 h. The media was aspirated and cells in each well were washed using 1 mL pre-warmed DPBS buffer. The cells were detached from the plate using a cell dissociation buffer (400 μL/well for 5 min in a humidified 5% CO_2_ incubator at 37°C). The cell suspension was collected in pre-labeled 1.5 mL centrifuge tubes and was centrifuged at 300× g for 3 min at 4°C. The supernatant was discarded, and the cell pellet was washed twice with ice-cold DPBS. The cell pellet was resuspended in 40 μL 1X RIPA (ice-cold 5X RIPA buffer was diluted in RNase free water) containing an aprotinin protease inhibitor, vortexed, and sonicated with a probe sonicator (Microson, Misonix Inc., Farmingdale, NY) for 10 pulses at 1-sec intervals twice on ice. The total protein content in cell lysates was measured using the Pierce BCA assay kit.

#### 2. BCA assay for total protein measurement

In a 96-well plate, a 2 μL cell lysate was diluted to 20 μL using 1X RIPA buffer. BCA standards (20 μL) from 25 to 1500 μg/mL was transferred to a 96-well plate in duplicate. A standard curve for total protein was plotted for each experiment. To each 20 μL sample, 180 μL BCA reagent mixture (reagent A: reagent B = 50:1) was added and incubated for 30 min at 37°C. The optical density of the samples was measured at 562 nm using a SYNERGY HTX multi-mode reader (BioTek Instruments, Inc., Winooski, VT).

#### 3. Western blotting

The proteins were separated based on molecular weight using a 4% stacking (top) gel and 12% running (bottom) SDS-polyacrylamide gel electrophoresis (SDS-PAGE). A 1.5 mm thick SDS- polyacrylamide gel with 15 lanes was prepared using a Bio-Rad assembly. Cell lysates containing 30, 40 or 50 μg total protein, the pre-mixed protein standards (250 kD-10 kD) and recombinant human BDNF protein (0.4 μg/lane) were mixed with 4x laemmli buffer and distilled water to prepare 20 μL sample volume per well. The samples were denatured at 95°C for 5 min using a block heater (Thermo Scientific) except for the protein ladder. The sample was loaded in the gel and ran at 120 V for 90 min using PowerPac Basic (Bio-Rad Laboratories, Inc.). The proteins were transferred to a 0.45 μm Nitrocellulose membrane at 75 V and 300 mA for 90 min using a transfer assembly (Fisher Scientific). The membrane was washed with 0.1% Tween 20-Tris buffer saline (T-TBS) buffer for an hour with periodic washes with fresh T-TBS. The membrane was then blocked with Odyssey blocking solution (Odyssey blocking buffer: T-TBS = 1:1) for an hour. The membrane was incubated with primary antibodies in Odyssey blocking solution at 4°C overnight (Tubulin: mouse anti-tubulin, 0.5 μg/mL; BDNF: rabbit polyclonal BDNF antibody, 2 μg/mL; P2X4R: mouse polyclonal, 1 μg/mL; and Iba1: rabbit polyclonal, 0.5 μg/mL). The membrane was washed with T-TBS and incubated with the respective secondary antibodies (Tubulin: donkey anti-mouse AF680; BDNF: goat anti-rabbit AF790; P2X4R: donkey anti-mouse AF790; and Iba1: goat anti-rabbit AF790 at a 1/30,000 dilution in Odyssey blocking solution) for an hour at room temperature on an orbital shaker. The membrane was again washed with T-TBS and imaged at 700 nm and 800 nm near-infrared channels using an Odyssey imager (LI-COR® Inc., Lincoln, NE) at intensity setting 5.

### In-cell western (ICW) for detecting P2X4R, Iba1, and BDNF protein expression

ICW was performed using the same methods as ICC that was described above. The only difference was the type of secondary antibodies and the nucleus staining marker. A mixture of secondary antibodies containing DRAQ5 5 mM at 1:10,000 dilution in blocking solution was used to stain the cell nucleus that emits fluorescence on the 700 nm channel. The following secondary antibodies were used at 1:30,000 dilution for ICW; BDNF and Iba1: goat anti-rabbit AF790; P2X4R: donkey anti-mouse AF790. The nucleus staining was detected using a 700 nm (red) channel, whereas target proteins were evaluated at the 800 nm (green) channel using an Odyssey imager (LI-COR® Inc., Lincoln, NE) using intensity setting 5 and plate height 4.0 mm.

### Cytocompatibility of LPS and ATP with SIM-A9 cells

#### 1. SIM-A9 cell viability using cell titer glo assay (ATP assay)

SIM-A9 (**passage number 6; P6**) cells were seeded in 96-well plates at a density of 16,500 cells/well in complete growth media. Following cell plating, the plates were incubated in a humidified 5% CO_2_ incubator at 37 ±0.5°C for 48 h. A stock solution of 1 mg/mL LPS was prepared in cell culture media. The cells were treated with media alone (control), 2.5, 10, 25, 100, 500 ng/mL, 1μg/mL, 25 μg/mL, and 50 μg/mL LPS in 200 μL of growth media. The cells were incubated for 2, 4, 6 and 24 h such that the total culturing time of cells (including treatment) prior to measurement would always remain the same at 72 h post-seeding. The Cell Titer Glo assay (ATP assay) was performed either immediately or 24 and 48 h post-LPS exposure adding a mixture of complete growth media: Cell Titer Glo reagent (60:60 μL). The plate was incubated at RT for 15 min in dark on an orbital plate shaker. A 60 μL mixture was transferred to 96-well white opaque plates to avoid loss of emitted light and prevent signal leakage among wells. Luminescence was measured using SYNERGY HTX multi-mode reader (BioTek Instruments, Inc., Winooski, VT) plate reader at 1 sec integration time.

#### 2. SIM-A9 cell viability using MTS assay

SIM-A9 (P6) at a cell density of 16,500 cells/well in complete growth media were seeded in 96-well plates. Following cell plating, the plates were incubated in a humidified 5% CO_2_ incubator at 37 ± 0.5°C for 48 h. A stock solution of 1mM ATP was prepared in cell culture media. The cells were treated with media alone (control), 25 nM, 100 nM, 500 nM, 1 μM, 50 μM, 100 μM, and 500 μM ATP in 200 μL of growth media. The cells were incubated for 4 and 24 h such that the total resident time of cells from the point of seeding to harvesting the cell lysates would remain the same i.e. 72 h. MTS assay was either performed immediately or 24 h and 48 h post-ATP exposure. DPBS was pre-warmed at 37°C prior to dissolving MTS and PMS. MTS solution at 2 mg/mL and PMS solution at 0.92 mg/mL in DPBS were freshly prepared and protected from light using an aluminum foil. MTS: PMS mixture was prepared at a 20:1 volume ratio. In each well, the old cell culture media was replaced with 100 μL cell culture media premixed with 20 μL of the MTS: PMS mixture. The plate was incubated at 37°C for 1–4 h. The absorbance was measured every hour for 4 h at 490 nm using a SYNERGY HTX multi-mode reader (BioTek Instruments, Inc., Winooski, VT). Cell viability of ATP treated SIM-A9 cells was calculated relative to the absorbance of the control, untreated cells.

### ATP-mediated SIM-A9 stimulation

SIM-A9 (**P8**) cells were seeded in three 24-well plates at a cell density of 100,000 cells/mL/well in complete growth media. SIM-A9 containing plates were incubated in a humidified 5% CO_2_ incubator 37 ±0.5°C for 48 h. A stock solution of 1 mM ATP in cell culture media was prepared. The cells in each well were treated with media alone (control), 1, 50, and 100 μM ATP in one mL of growth media. The cells were incubated for 2, 4, 6 and 24 h such that the cells were further subsequently cultured for a total of 72 h (including ATP exposure times) prior to harvesting the cell lysates. Post-72 h, the cells were washed with 1x PBS and dissociated using a non-enzymatic cell-dissociation buffer containing 1 mM EDTA + 1 mM EGTA +1 mg/mL glucose. The cells were collected in 15 mL centrifuge tubes and washed twice with ice-cold PBS. The cells were lysed with 1X RIPA buffer and sonicated with a probe sonicator for 10 pulses twice at 1 s intervals. Cell lysates were stored at -80°C until performing a BCA assay for total protein measurement followed by western blotting analysis.

### Quantification and statistical analysis

Band density in western blots and signal intensity in ICW were quantified using ImageStudio Lite 5.2 software. Western blot data were normalized with alpha-tubulin levels and compared with the band density of control, untreated cells. ICW data were normalized with nucleus staining and non-specific secondary antibody staining followed by comparison with control, untreated cells. Data in figures indicate mean ± standard deviation and ‘n’ values indicate the number of replicates in each group. GraphPad Prism 8.2 software was used for statistical analysis. Mean comparison between control and treatment groups in cell viability studies were performed using one-way ANOVA followed by Dunnett’s multiple comparison tests. For western blot and ICW, statistical significance between control and treatment groups was determined using two-way ANOVA followed by Tukey’s multiple comparison tests. p<0.05 was considered statistically significant.

## Results

### 1. SIM-A9 cells express the microglia marker, Iba-1, the purinergic receptor, P2X4R and the neurotransmitter modulator, BDNF

#### Immunocytochemistry (ICC)

**Fig 1:**
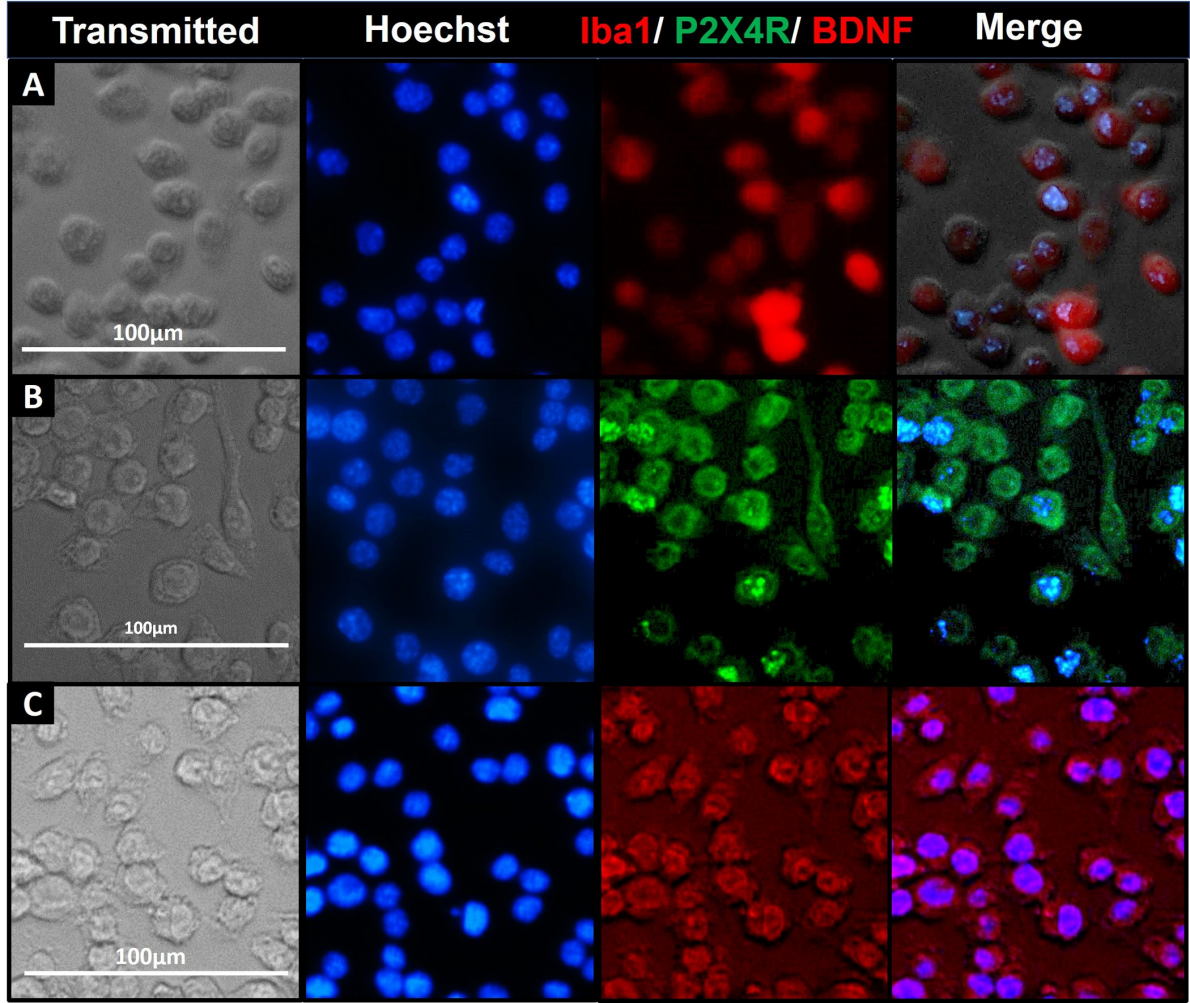
Iba1, BDNF and P2X4R detection using immunocytochemistry (ICC). ICC study to detect the expression of microglia-specific markers (Iba1, **A**) and proteins involved in the signaling pathway initiating pain hypersensitivity (P2X4R, **B**; and BDNF, **C**) in SIM-A9 cell line. SIM-A9 cells (Passage no. 9; P9) were cultured in a 96-well plate in complete growth media for 48 h. The cells were fixed with 4% PFA followed by immunostaining for Iba1, P2X4R, and BDNF. The cells were captured under phase contrast setting (Transmitted) and nuclei were counterstained using Hoechst 33342. The signals for P2X4R were imaged under the green fluorescence channel (excitation 470/22 nm and emission 510/42 nm) whereas Iba1 and BDNF were imaged under red fluorescence channel (excitation 531/40 and emission 593/40). Similar observations were noted in triplicate wells/group in three independent experiments. Scale bar = 100 μm. All images were cropped at the same scale using Adobe Photoshop CC 19.1.9 for clarity and conciseness of the presentation. Full-length images of a representative set (**B**) is presented in **S1 Fig**.

SIM-A9 cells were examined for subcellular expression of the microglia marker protein; Iba1 and P2X4R and BDNF, key molecules involved in the signaling axis that initiates pain hypersensitivity. The above proteins were detected in immunostained cells under phase contrast, DAPI (nucleus), RFP (Iba1 and BDNF), and GFP (P2X4R) channels. ICC staining showed that SIM-A9 cells cultured for 48 h in complete growth media expressed Iba1. Almost every cell in the frame showed notable fluorescence associated with intracellular Iba1 expression (**Fig 1A, red**). In addition, SIM-A9 cells were also stained for P2X4R present in the cytosol [20, 32]. We observed notable fluorescence signals, confirming P2X4R expression (**Fig 1B, green**). In each cell, the presence of red fluorescence confirmed BDNF expression in the cytosol (**Fig 1C, red**). Collectively, the ICC results confirmed the expression of Iba1, P2X4R, and BDNF expression in SIM-A9 cells.

#### Western blotting

**Fig 2:**
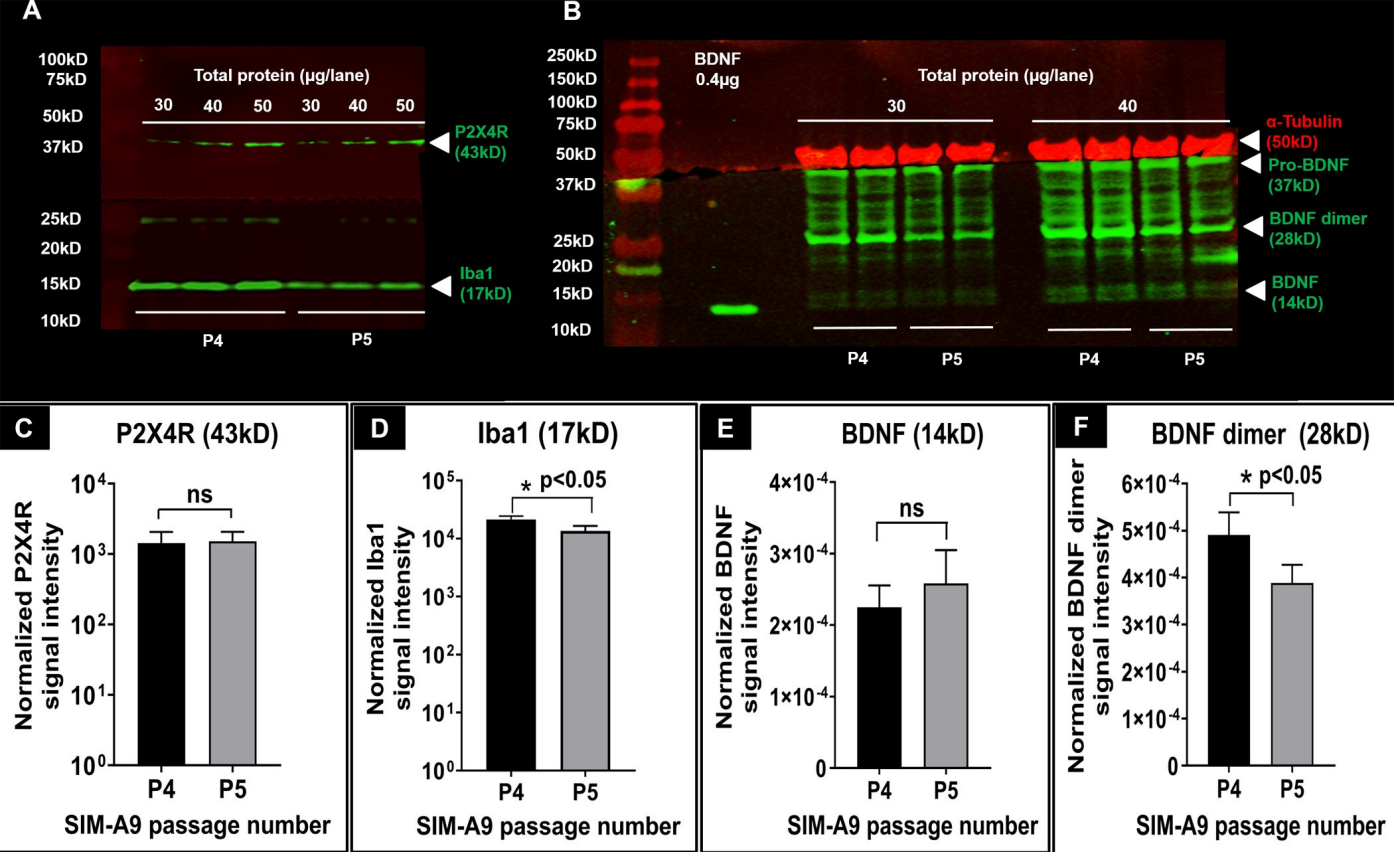
P2X4R, BDNF and Iba1 detection using western blotting. Western blot of P2X4R and Iba1 proteins (**A**), and α-tubulin, BDNF and its isoforms in SIM-A9 cell lysates (**B**) collected from different passages. In each blot, the series of bands on the left shows the protein ladder. For P2X4R and Iba1 (**A**), cell lysates collected from P4 and P5, at 30, 40, and 50μg/lane total protein were used to detect P2X4R (green, 43kD) and Iba1 (green, 17kD) on the 800 nm channel. The signals at about 25 kD are due to the non-specific reactivity of secondary antibody. For α-tubulin and BDNF (**B**), cell lysates at 30 and 40 μg/lane total protein were electrophoresed to identify α-tubulin (50 kD), BDNF monomer (14 kD), BDNF dimer (28 kD), and pro-BDNF (37 kD) on the 700 nm and 800 nm channels, respectively. The blots were scanned using an Odyssey imager at intensity setting 5 and processed using ImageStudio 5.2 software. Each sample was run in triplicates (**A**) or duplicates (**B**). **C-F**. Densitometry analysis of P2X4R, Iba1, BDNF, and BDNF dimer bands was performed using ImageStudio 5.2 software. Signal intensities of P2X4R (**C**) and Iba1 (**D**) bands were normalized for protein loading. Signal intensities of BDNF (**E**) and BDNF dimers (**F**) were first normalized to α-tubulin and then to the amount of protein loaded. Statistical analysis between P4 and P5 was performed by unpaired t-test and Welch’s correction using GraphPad Prism 8.1.2. Asterisks indicate statistically significant differences (* p<0.05). Blots were merged and cropped for the clarity and conciseness of the presentation. Full-length blots are presented in **S3A and S3B Fig, raw blots A and B**.

While ICC allowed us to study their subcellular localization, we confirmed protein expression in the total cell lysates using western blotting. Prior to using SIM-A9, we determined BDNF expression levels in U-87MG (human glioblastoma/astrocytoma cell line) to see if we could modulate BDNF expression in these cells using LNP-siBDNF formulations. Once we procured SIM-A9 cells, we shifted our focus to this microglial cell line. **S2A Fig** showed that U-87MG at P131 and 132 expressed BDNF (14 kD), BDNF dimer (28 kD), pro-BDNF (37 kD). **S2B Fig** depicted Iba1 expression in both SIM-A9 and U-87MG cell lysates. Iba1 expression was lower in the U-87MG cell line compared to SIM-A9 cells which is likely due to the differences in cell-type-specific expression of Iba1 in cells of astrocytic vs. microglial origin. P2X4R (43 kD) was expressed in SIM-A9, but not in U-87MG cell line. SIM-A9 cells were lysed and the expression of Iba1, P2X4R, and BDNF expression at their characteristic molecular weights was confirmed using western blotting. We confirmed the expression of the above proteins in lysates harvested from two subsequent passages (P). SIM-A9 cell lysates from P4 and P5 were loaded at 30, 40, and 50 μg in each lane. The blot (**Fig 2A**) showed P2X4R and Iba1 expression (green signals from the 800 nm channel) in SIM-A9 cell lysates at their characteristic molecular weights of 43 kD and 17 kD, respectively. In **Fig 2A**, we noted increased band densities for P2X4R and Iba1 expression that correlated with the increased loading amounts. Densitometry analysis was performed using Image studio 5.2 and the results are shown in **Fig 2**. Signal intensities of Iba1 and P2X4R bands in **Fig 2A** were normalized for protein loading and a comparative evaluation was performed between P4 and P5 using unpaired parametric t-test and Welch’s correction assuming no equal variance between two groups. There was not a statistically significant difference between P4 and P5 P2X4R expression (**Fig 2C**), whereas a small yet significant difference (p = 0.032) between P4 and P5 Iba1 expression (**Fig 2D**) was observed.

Furthermore, we detected the expression of BDNF in P4 and P5 cell lysates and used α-tubulin as an endogenous loading control. A single band of α-tubulin was detected at 50 kD (**Fig 2B**, red). The expression of α-tubulin in SIM-A9 did not vary with the loading amount. A possible reason could be the signal saturation of α-tubulin at 30 μg loading that likely did not allow us to discern differences at higher amounts of protein loading. We detected the following three BDNF isoforms: pro-BDNF, BDNF dimer and monomer at 37, 28, and 14 kD, respectively. Human recombinant BDNF (14 kD) was used as a standard for BDNF monomer identification. SIM-A9 cells expressed considerable amounts of pro-BDNF (37 kD) and BDNF dimer (28 kD). Densitometry analysis was performed and signal intensities of BDNF and BDNF dimers were first normalized to α-tubulin and then to the amount of protein loaded. Statistical analysis between P4 and P5 was performed by unpaired t-test and Welch’s correction. There was no significant difference in BDNF expression at P4 vs. P5 (**Fig 2E**), whereas BDNF dimer expression in **Fig 2F** was slightly lower at P5 (p-value 0.018).

Taken together, we confirmed the expression of Iba1, P2X4R, and BDNF in SIM-A9 cells using western blotting. A total protein loading of 50 μg produced observable band densities for P2X4R and BDNF while loading a lower amount (30 μg) showed only intense expression of the housekeeping proteins Iba1 and α-tubulin.

#### In-cell western

In-cell western (ICW) is a powerful technique with a high sensitivity that allows multiplexing samples compared to the laborious western blotting and was used to confirm the expression of Iba1, P2X4R, and BDNF in SIM-A9 cells. In order to establish an ICW assay for detecting the above proteins, preliminary studies were performed by optimizing secondary antibodies and their dilution, fixative agents and incubation time, and the effect of including a cell permeabilizer in the protocol (**S4 and S5 Figs**). The detailed optimization process is discussed below **S4 and S5 Figs** in the Supporting Information file. Briefly, primary rabbit antibodies for Iba1 and BDNF at 1:500 dilution followed by counterstaining with goat anti-rabbit AF790 (1:700) showed reproducible and robust expression of intracellular Iba1 and BDNF **(S4 Fig)**. The expression of P2X4R was examined in fixed cells **(S5A Fig)** versus live (without fixatives) cells **(S5B Fig)**, and with or without a permeabilizer (0.3%v/v Triton X-100) in the blocking and antibody solutions. Without fixatives, cells were washed off during multiple washing and blocking steps that are involved in an ICW experiment (**S5B Fig**). Interestingly, we did not detect surface P2X4R expression in resting or activated SIM-A9 in the absence of a permeabilizer (**S5A Fig**). In contrast, considerable fluorescent signals were observed in permeabilized cells suggesting the presence of P2X4R in the cell cytoplasm.

**Fig 3:**
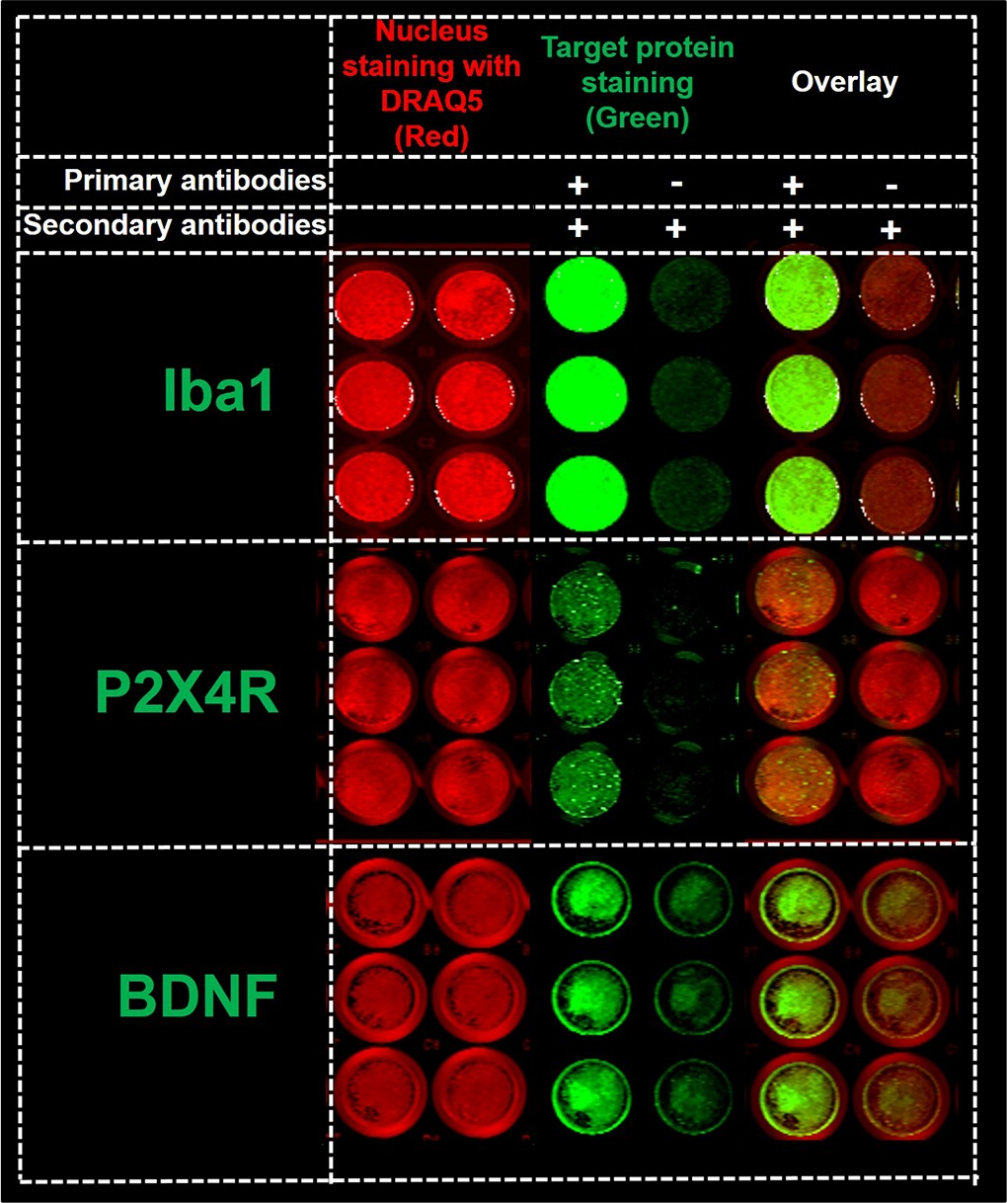
In-cell western for Iba1, P2X4R and BDNF detection. The detection of Iba1, P2X4R, and BDNF expression (green fluorescence, 800 nm channel) and cell nucleus (red fluorescence, 700 nm channel) in 96-well plates using In-cell western (ICW). SIM-A9 cells in 96-well plates were fixed with 4% PFA and were treated using a Li-COR Odyssey blocking solution. Each protein was analyzed in two columns. For each group (nuclear staining, target protein staining, and overlay), the cells in the left columns were immunostained with Iba1, P2X4R, or BDNF primary and secondary antibodies (+/+), whereas the primary antibodies were omitted in the right columns (-/+). All wells were incubated with AF790 secondary antibodies (1:700 dilution) and DRAQ5 (nucleus stain, 1:3000) prior to scanning using a Li-COR Odyssey near-infrared imaging system at intensity setting 5 and plate height 4.0 mm. Three independent experiments were performed with n = 3 wells per group. ICW images of 96-well plates for each protein were merged and cropped for the clarity and conciseness of the presentation. Full-length scans are presented in **S4A–S4C Fig**.

After optimizing the ICW protocol, SIM-A9 cells were characterized for Iba1, P2X4R, and BDNF expression (**Fig 3**). The optimized ICW parameters resulted in strong fluorescence signals corresponding to Iba1, intracellular (but not surface) P2X4R, and BDNF expression with a minimum background signal.

### 2. Lipopolysaccharide was cytotoxic whereas Adenosine triphosphate was cytocompatible with SIM-A9 cells

#### Cytotoxicity of lipopolysaccharide

Lipopolysaccharide (LPS) is an inflammatory stimulator widely used in *in-vitro* [26, 33, 34] and preclinical models [35, 36] to activate various cell types [26]. It has been demonstrated that cell stimulation is dependent on LPS concentration and incubation time [26, 33, 37]. In order to determine the safe concentration and exposure time for SIM-A9 cells, cells were incubated with LPS concentrations ranging from 2.5 ng/mL to 50 μg/mL for times varying from 2 to 24 h. The effect of LPS on SIM-A9 cell viability was examined either immediately, 24, or 48 h post-LPS treatment using a Cell Titer Glo assay (denoted henceforth as ATP assay). Our goal was to identify treatment conditions at which the cell viability post-treatment was ≥90%.

**Fig 4:**
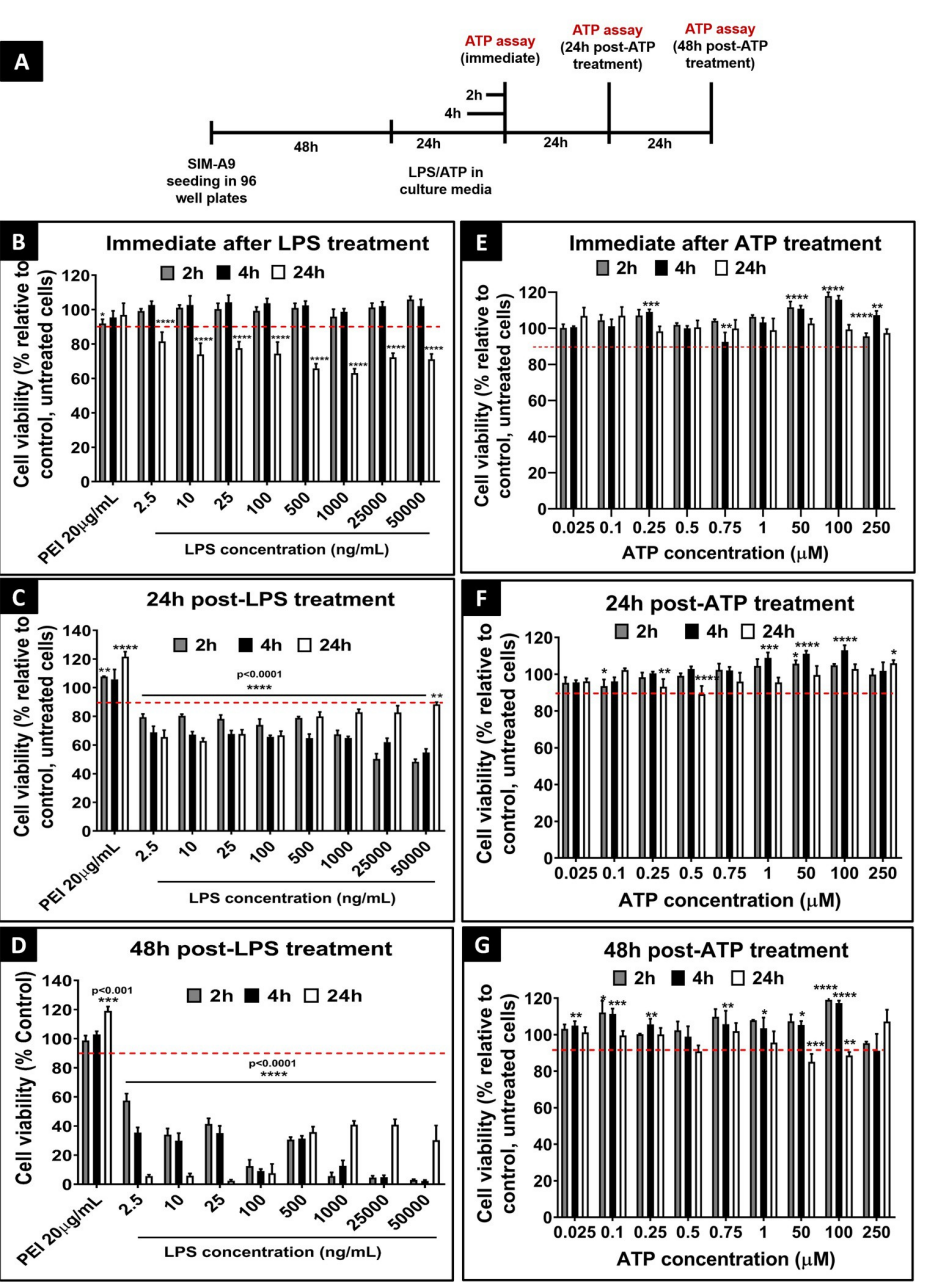
Cell viability study using cell titer glo assay (ATP assay). Effect of LPS (**B, C,** and **D)** and ATP (**E**, **F**, and **G**) exposure on the cell viability of SIM-A9 microglia cells (P5 and P6) immediately (**B** and **E**), 24 h post-treatment (**C** and **F**), and 48 h post-treatment (**D** and **G**) using ATP assay. The experimental scheme is shown in **A**. SIM-A9 cells were cultured in a 96-well-plate at 16,500 cells/well for 48 h. Cells were exposed to 2.5 ng/mL to 50 μg/mL LPS for 2, 4 or 24 h. Cells were treated with 25 nM to 250 μM ATP for 2, 4 or 24 h. The cell viability was evaluated using an ATP assay either immediately, 24 or 48 h post-LPS or ATP treatment. The cell viability of treated cells was calculated relative to control, untreated cells. PEI at 20 μg/mL was used as a positive control. Statistical analysis was performed using GraphPad Prism 8.1.2. Asterisks indicate significant differences (**** p<0.0001, *** p<0.001, ** p<0.005, * p<0.05) compared to the control. The data is representative of two independent experiments and is presented as mean ± standard deviation (SD) of at least n = 4 wells per group.

An experimental scheme is depicted in **Fig 4A**. Branched polyethyleneimine (PEI, 20 μg/mL, 25 kD), a synthetic polycation, was used as a positive control. SIM-A9 cells incubated for 2 and 4 h showed >90% cell viability (----; red dashed line) when the cell viability was measured immediately after LPS treatment (**Fig 4B**). However, cells incubated for 24 h at LPS concentrations as low as 2.5 ng/mL exhibited a significant (p<0.0001) reduction in cell viability. The viability was further significantly (p<0.0001) compromised in cells treated with higher LPS concentrations. Twenty-four hours post-LPS treatment (**Fig 4C**), the entire range of tested LPS concentrations showed a significant (p<0.0001) reduction in cell viability ranging from 20 to nearly 40%. The recovery of the %viability values in cells treated at higher LPS concentrations 24 h post-exposure may be due to the regeneration of cells during the post-exposure period. Furthermore, groups measured 48 h post-LPS treatment **(Fig 4D)** showed more than a 50% reduction in SIM-A9 cell viability compared to control, untreated cells. To summarise, the data suggested that LPS was cytotoxic to SIM-A9 cells at concentrations as low as 2.5 ng/mL even for the shortest evaluated incubation time (2 h). Notably, positive control (PEI, 20 μg/mL) in the above experiments did not show a significant reduction in cell viability. A possible reason is due to the resilience of SIM-A9 cells treated at 20 μg/mL PEI concentration. Therefore, in the subsequent experiments (**S6 Fig**), we tested higher PEI concentrations (40, 50, and 100 μg/mL) as a positive control. We additionally performed a MTS assay confirming the immediate- (**S6B Fig**), 24 (**S6C Fig**) and 48 h post-treatment (**S6D Fig**) viability of LPS-treated SIM-A9 cells. SIM-A9 cells were incubated with varying concentrations of LPS in the complete growth medium for 4 (black bars) and 24 h (white bars). The viability was above 90% when measured immediately and 24 h-post LPS treatment for 4 and 24 h exposures (**S6B and S6C Fig**). As shown in **S6D Fig**, the viability of cells exposed to 2.5 ng/mL LPS for 4 h followed by a 48 h regeneration showed a statistically significant (p<0.01) reduction in cell viability (75.5±0.4%) compared to the control. The viability further declined to 13.9±3.3% after 24 h incubation of 2.5 ng/mL LPS. Noteworthy, the MTS assay detected the drastic reduction in cell viability after LPS treatment only after 48 h-post LPS treatment, whereas the ATP assay detected the LPS-mediated cytotoxicity as early as 24 h post-LPS incubation. It is likely that the ATP assay sensitively captures the ATP decreases during early-stage apoptosis in injured SIM-A9 cells. LPS-mediated SIM-A9 cytotoxicity was further confirmed using trypan blue staining studies **(S7 and S8 Figs)** done using a Countess II counter (Invitrogen, Singapore) and microscopic images **(S9 and S10 Figs)** obtained from an EVOS microscope. As shown in **S8 Fig**, images of cells treated with 2.5 ng/mL LPS for 24 h depicted a complete loss in cells compared to the control group. In **S10 Fig**, cells looked healthy, adhered fully and were homogeneously distributed in a control well at 48 h post-treatment. On the other hand, in LPS-treated wells, the cells were semi-adhered, clustered and showed unequal-density throughout the well, reflecting cell stress and toxicity.

### Cytocompatibility of adenosine triphosphate

Peripheral nerve injury stimulates the release of ATP from spinal dorsal horn neurons within the spinal cord [21, 38]. Extracellular ATP activates P2X4 receptors on microglia and plays a critical role in the signaling pathway leading to the initiation of pain hypersensitivity and the production of tactile allodynia in neuropathic pain [21, 38]. Therefore, we tested the cytocompatibility of ATP with SIM-A9 cells. As shown in **Fig 4A**, SIM-A9 cells were incubated with ATP at concentrations ranging between 25 nM to 250 μM for 2, 4 and 24 h and the cytocompatibility was determined using ATP assay. ATP at 25 nM to 250 μM was well tolerated by the cells (viability >90%) under the tested experimental conditions (**Fig 4E** and **4F**). The viability measured 48 h post-treatment after cells were exposed to ATP for 2 and 4 h was >90% (**Fig 4G**). Notably, cells exposed to 50 and 100 μM ATP for 24 h showed a slight decrease in cell viability to ca. 87%, but that was still close to our acceptable value of 90%. Our results suggested that 50 and 100 μM ATP exposure for 24 h may cause observable toxicity 48 h post-treatment. Therefore, we did not incubate cells for more than 24 h with ATP concentrations > 50 μM. In addition, cell viability of SIM-A9 cells was determined using MTS assay either immediately, 24 or 48 h post-ATP treatment. As shown in **S6A Fig**, SIM-A9 cells were incubated with ATP at concentrations ranging between 25 nM to 250 μM for 4 and 24 h. Immediately after the ATP exposure, the cell viability of groups treated for 4 and 24 h was about 100% (**S6E Fig**) whereas PEI exposure resulted in a concentration and time-dependent reduction in cell viability (p<0.0001). After 4 h incubation, PEI at 50 and 100 μg/mL showed nearly a 20% and 70% reduction in cell viability respectively, while at 24 h incubation, it showed about 50% and 80% reduction in cell viability. We studied the effect of ATP exposure on cell viability at 24 **(S6F Fig)** and 48 h **(S6G Fig**) post-treatment. No significant reduction in cell viability was noted across the entire range of ATP concentrations ranging from 25 nM to 250 μM. In conclusion, LPS was cytotoxic to SIM-A9 cells exposed at concentrations of ≥ 2.5 ng/mL for 2 h or longer. ATP was cytocompatible with SIM-A9 cells for 24 h at the highest tested concentration of 250 μM ATP.

### 3. ATP-mediated stimulation of SIM-A9 cells led to overexpression of Iba1 and BDNF

#### a. Western blot analysis

ATP plays a critical role in developing PNI-induced neuropathic pain hypersensitivity [31]. ATP stimulates P2X4R on microglia leading to its activation and overexpression of intracellular BDNF, a key modulator of pain hypersensitivity [31]. In order to establish an *in vitro* microglia activation model, we exposed SIM-A9 microglia cells to different ATP concentrations for various times and studied changes in the microglial activation marker, Iba1, and proteins (P2X4R and BDNF) involved in this signaling axis. First, we confirmed the effect of serum in the treatment medium on Iba1 and BDNF expression. The cell culture condition for SIM-A9 cells are identical during “resting”, “control” and “unstimulated”—cells were simply cultured in the complete growth medium. Western blot studies in S11 Fig were performed using 50 μM ATP dissolved in serum-containing or serum-free medium and incubated for the cells for 2, 4 and 6 h. ATP treatment in serum-containing medium demonstrated a considerable increase in BDNF, BDNF dimer and Iba1 expression at 2 and 4 h compared to the control. However, ATP 50 μM treatment in serum-free medium showed a considerable increase in BDNF and Iba1 only at the 2 h time point and demonstrated a considerable decrease in the expression levels of all proteins at 4 and 6 h. Based on these results, we infer that serum in the treatment media provides cell nourishment allowing optimal protein expression and shows only transient effects of ATP-mediated stimulation.

**Fig 5:**
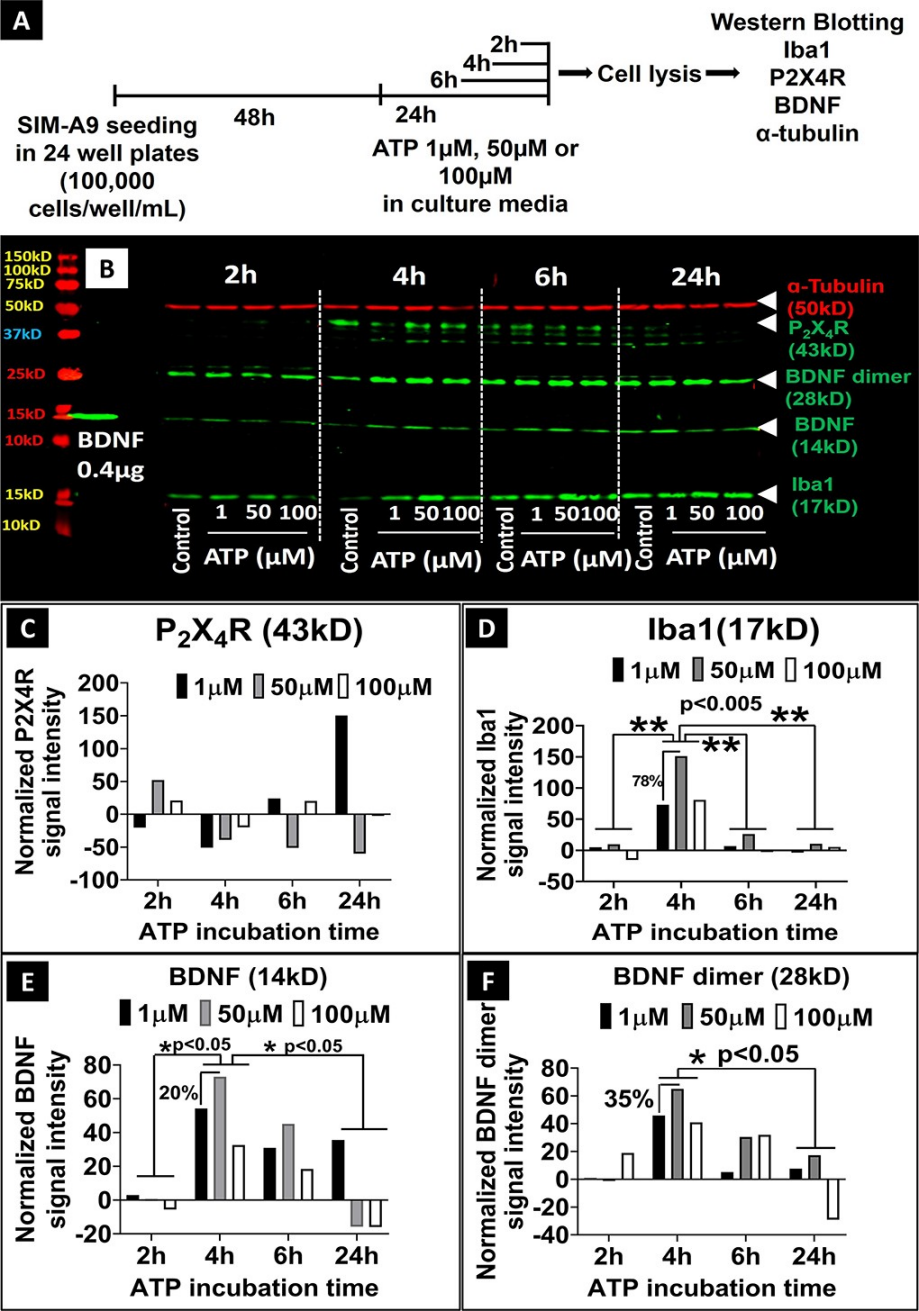
Effect of ATP on the expression of Iba1, P2X4R, BDNF, and α-tubulin proteins in SIM-A9 microglia cells analyzed using western blot. **A**) SIM-A9 cells (P6) were cultured for 48 h and exposed to 1, 50, and 100 μM ATP for 2, 4, 6 and 24 h, then lysed immediately after the treatment for subsequent analysis. **B**) For P2X4R and BDNF evaluation, cell lysates were loaded at 50 μg in each lane, and the loading amount for Iba1 and α-tubulin was 30 μg/lane. Human recombinant BDNF protein (14 kD) was loaded at 0.4 μg/lane as a positive control. ATP concentration and time-dependent modulation of the levels of α-tubulin (50 kD, red), P2X4R (43 kD), BDNF (14 kD), BDNF dimer (28 kD) and Iba1 (17 kD) was studied after scanning the blots at 700 nm and 800 nm channels using Odyssey imager at intensity setting 5. **C**-**F**. Densitometry analysis of P2X4R, Iba1, BDNF, and BDNF dimer bands was performed using ImageStudio 5.2 software. Normalized signal intensity in Y-axis represents a normalization of protein signal intensity to, first, α-tubulin signal intensity followed by normalization to the signal intensity of control/untreated cells at each time point. Note: **C-F** demonstrates the quantification of the western blot shown in **Fig 5B**. It should be noted that the experiment was performed three independent times and the densitometry analysis for representative proteins, BDNF and Iba1, are shown in **S12 Fig**. Statistical analysis between specified groups was performed using GraphPad Prism 8.1.2. Asterisks indicate statistically significant differences (** p<0.005, * p<0.05). Blots were merged and cropped for the clarity and conciseness of the presentation. Full-length blots are presented in **S13 Fig**.

As shown in **Fig 5A**, SIM-A9 cells were cultured for 48 h and incubated with 1, 50 and 100 μM ATP in the complete (serum-containing) growth medium for 2, 4, 6 and 24 h. Cell lysates collected from untreated cells at each of the time points were used as a control. Cells exposed to ATP were lysed immediately after the defined treatment period and the total protein content was measured. Equal quantities of total protein were analyzed using SDS-PAGE to evaluate the effect of ATP exposure on P2X4R, BDNF, and Iba1 and α-tubulin expression (loading control).

Western blot image in **Fig 5B** shows α-tubulin, P2X4R, BDNF, BDNF dimer, and Iba1 expression immediately after ATP exposure at the indicated concentrations and times. We confirmed the presence of P2X4R at its characteristic molecular weight of 43 kD in SIM-A9 cells. The low levels of P2X4R expression 2 h time point made those bands hard to visualize in the blot. Densitometry analysis in **S1 Table** lists the P2X4R protein band signal intensities demonstrating that 50 μM ATP exposure for 2 h increased P2X4R expression by 50% compared to the control group (**Fig 5C,** normalized to α-tubulin). At 4 h, the control group showed maximum P2X4R expression which was decreased in ATP (1, 50 and 100 μM)-treated SIM-A9 cells. SIM-A9 cells treated with ATP at 1 and 100 μM for 6 h showed nearly 10% higher P2X4R expression compared to the control group. Lastly, at 24 h, the expression level of P2X4R in the control group was lower compared to the levels at 4 and 6 h. In conclusion, SIM-A9 expressed P2X4R considerably at 4 and 6 h time points and showed greater expression in the control group compared to the ATP-treated cells.

The effect of ATP concentration and incubation time on Iba1 expression was compared to the control, untreated cells. At 2 h, 50 μM ATP showed a ca. 10% increase in Iba1 expression (**Fig 5D**). At 4 h, 50 μM ATP showed about a 150% increase in Iba1 expression compared to the control group which was nearly 78% higher than cells treated with 1 and 100 μM ATP. At the 6 and 24 h time points, the Iba1 expression was diminished significantly (p<0.005) compared to 4 h ATP and the control groups as well. In conclusion, the highest Iba-1 expression was noticed in SIM-A9 cells activated with 50 μM ATP for 4 h.

BDNF exists as three different isoforms in microglial cells: pro-BDNF (37 kD), dimer (28 kD) and mature (14 kD). BDNF expression in untreated SIM-A9 cells was confirmed in an earlier experiment (**Fig 2**). The goal of this experiment to evaluate the ATP-stimulated changes in BDNF expression using western blotting. As observed in **Fig 5B and 5E**, at 2 h, there were no changes in BDNF expression in 1 and 50 μM ATP-treated SIM-A9 cells, however, BDNF dimer showed a 20% increase when treated with 100 μM ATP (**Fig 5F**). At 4 h, there was a significant (p<0.05) increase in BDNF expression levels in ATP-treated groups. We noticed ca. 50%, 70%, and 40% increases in the expression of BDNF dimer levels when the cells were exposed to 1, 50 and 100 μM ATP, respectively. Cells treated for 6 h showed higher a BDNF expression compared to the control group, but lower than those treated for 4 h. In cells treated for 24 h, BDNF dimer and monomer expression decreased significantly (p<0.05) compared to those treated for 4 and 6 h. Overall, the greatest BDNF dimer and monomer expression were observed in cells treated with 50 μM ATP for 4 h. Noteworthy, ATP exposure showed similar effects on Iba1 expression.

Endogenous α-tubulin expression was evaluated as a loading control. Notably, there were no considerable changes observed in α-tubulin expression (except in the group treated with 100 μM ATP for 4 h) indicating that ATP treatment did not affect intracellular α-tubulin levels.

#### b. ICW analysis

**Fig 6:**
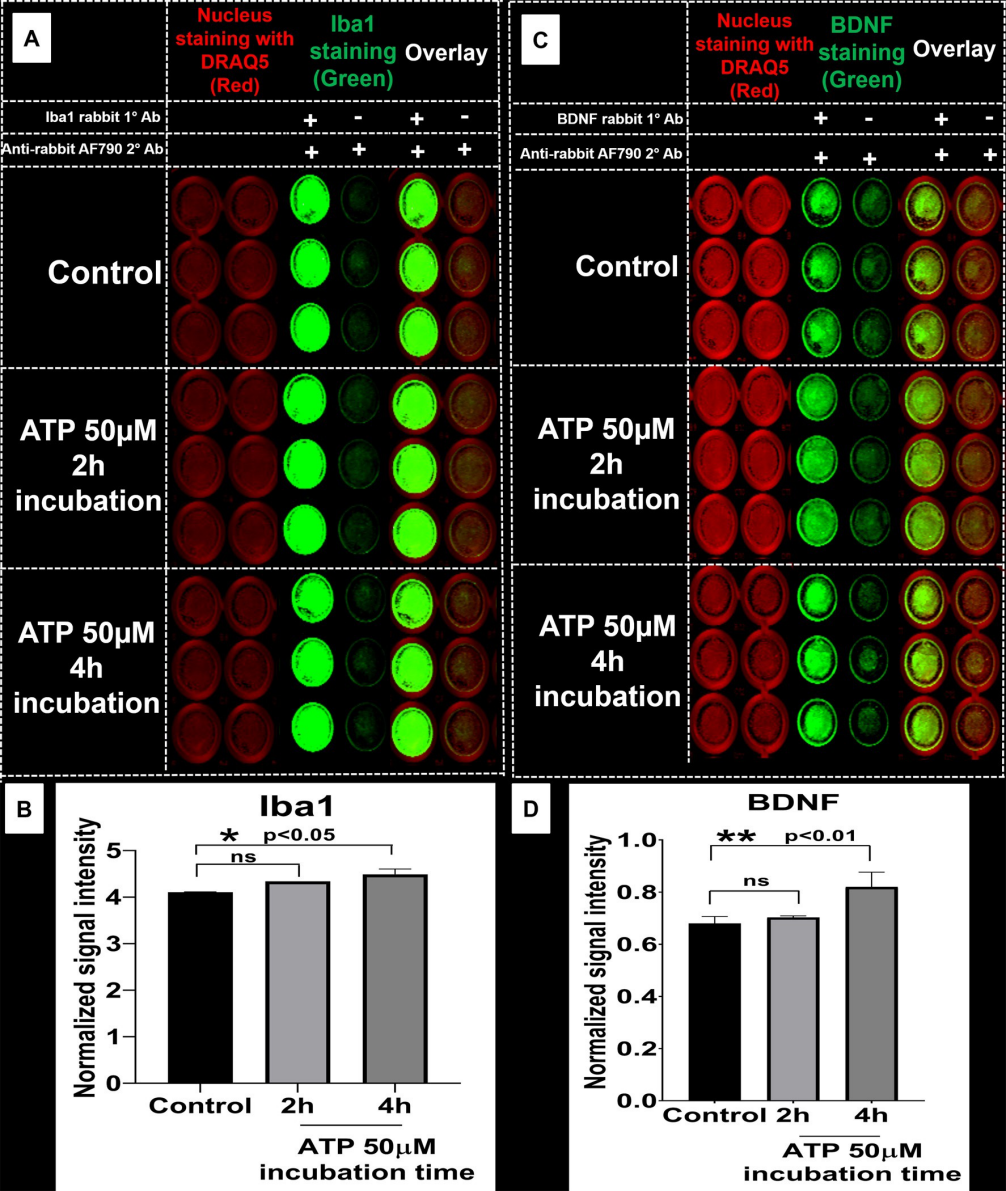
In-cell western analysis of the effect of ATP stimulation on Iba1 and BDNF expression. **A**) SIM-A9 microglia cells (P8) were cultured for 48 h and exposed to 50 μM ATP for 2 and 4 h. Cells were fixed with 4% PFA, then treated with blocking solution and Iba1 protein was stained using rabbit Iba1 (1:500) and anti-rabbit AF790 (1:700, green). **C**) BDNF was stained using rabbit BDNF (1:500) and anti-rabbit AF790 (1:700, green). Nucleus was stained with DRAQ5 (red). Non-specific binding of secondary antibodies was accounted for by omitting primary antibodies in each group. **B** and **D**) Densitometry analysis of each plate (Iba1 and BDNF) was performed using ImageStudio 5.2 software. Normalized signal intensity in Y-axis represents the normalization of protein signal intensity to nuclear staining followed by subtraction of non-specific signal intensity (-/+, no 1°, only 2° Ab). Statistical analysis between specified groups was performed using GraphPad Prism 8.1.2. Asterisks represent statistically significant differences (* p<0.05). Data are presented as mean ± SD of n = 3 samples. ICW images of 96-well plates for each protein were merged and cropped for the clarity and conciseness of the presentation. Full-length blots are presented in **S14 Fig**.

The effects of ATP stimulation on Iba1 and BDNF expression was studied using ICW. We examined the immediate changes in Iba1 and BDNF expression in SIM-A9 cells treated with 50 μM ATP for 2 and 4 h (**Fig 6**). The effect of ATP concentration and incubation time on Iba1 is presented in **Fig 6A**. We did not visually observe the ATP-mediated changes in Iba1 expression, however, the statistical analysis of the average signal intensity of Iba1 (obtained after normalization of protein signal intensity to nuclear staining followed by subtraction of non-specific secondary antibody signal intensity) showed that there was a statistically significant increase in Iba1 expression at the 4 h timepoint (p<0.05) (**Fig 6B**).

BDNF expression in the control group was confirmed by the presence of green fluorescence in **Fig 6C**. Furthermore, to evaluate the ATP-stimulated BDNF modulation, cells were treated with 50 μM ATP for 2 and 4 h. The values obtained after normalization of protein signal intensity to nuclear staining followed by subtraction of non-specific secondary antibody signal intensity are shown in **Fig 6D**. The one-way ANOVA performed on BDNF densitometry data suggested that ATP 50 μM exposure resulted in significantly (p<0.01) higher BDNF expression at 4 h compared to the control. There was no statistically significant difference observed in Iba1 and BDNF expression at 2 h using ICW. Likely, the signal intensity saturation in the control group did not allow us to discern the subtle differences in Iba1 and BDNF expression at 2 h. In addition, as shown in **S15B Fig**, BDNF expression was either decreased in cells treated with LPS at 2.5 ng/mL for 4 h (p≤0.05) or remained unaltered at 100 ng/mL exposure (p = 0.32). In summary, SIM-A9 showed increased BDNF expression when treated with 50 μM ATP for 4 h.

#### c. Immunocytochemistry

**Fig 7:**
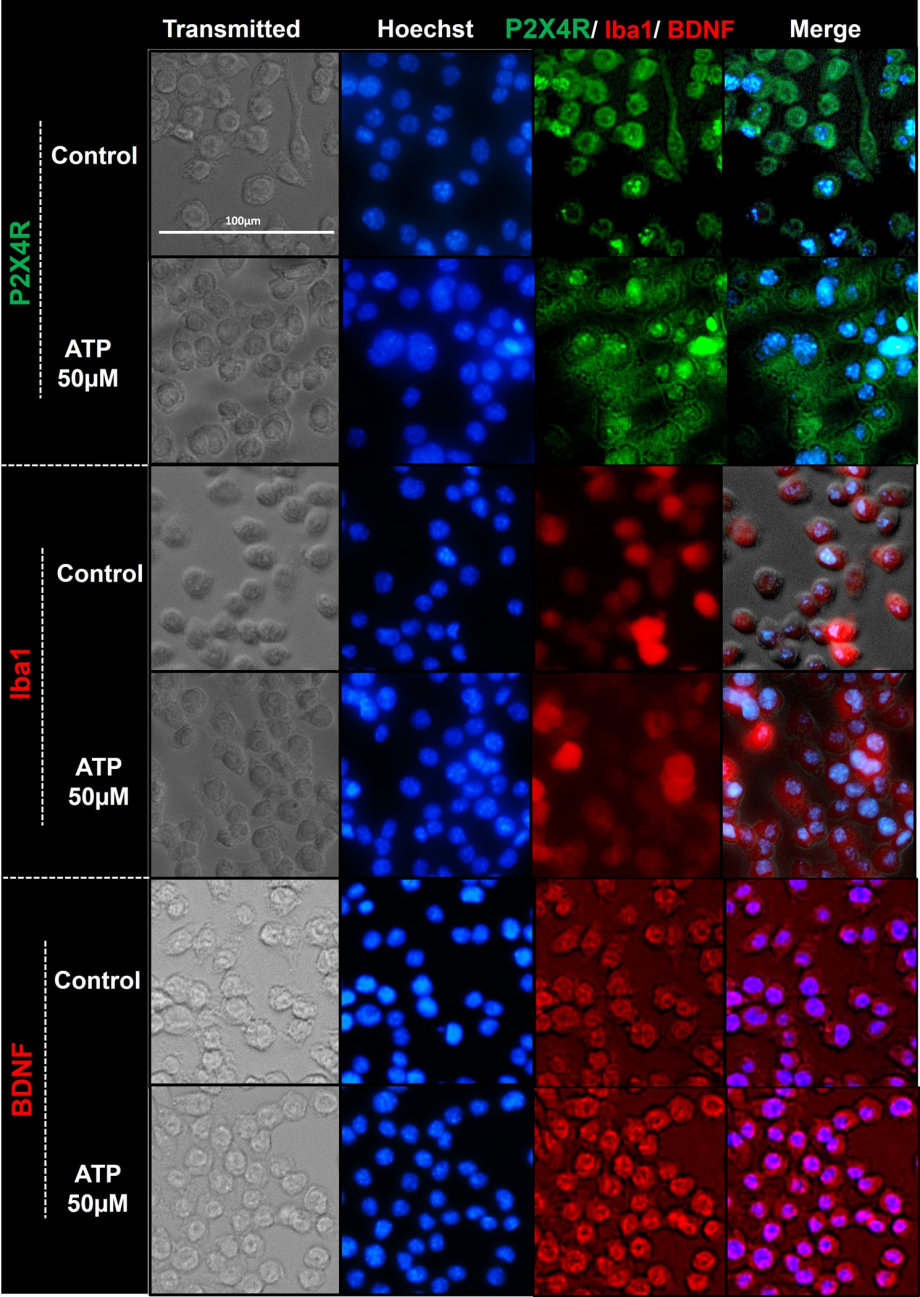
Immunocytochemistry images depicting ATP-mediated modulation of P2X4R, Iba1, and BDNF expression in SIM-A9 microglia cells. SIM-A9 cells (P8) were cultured for 48 h and incubated with 50 μM ATP for 2 h. Control, untreated cells, and treated cells were fixed with 4% PFA, blocked to prevent non-specific antibody binding, and immunostained with mouse-P2X4R (1:250), rabbit-Iba1 (1:500), and BDNF (1:500) primary antibodies, respectively. P2X4R was counterstained with anti-mouse AF555 (green), Iba1 and BDNF with anti-rabbit AF488 (red), and the nucleus was stained with Hoechst 33342 dye (blue). Images were captured under the phase-contrast setting (Transmitted), DAPI (blue), green or red channels, and merged in the overlay image. Triplicate samples were analyzed in two independent experiments. Scale bar = 100 μm. All images were cropped at the same scale using Adobe Photoshop CC 19.1.9 for clarity and conciseness of the presentation. The full length of a representative phase-contrast image for P2X4R is presented in **S1 Fig**.

ATP-mediated changes in the intracellular expression of P2X4R, Iba1, and BDNF expression were visually confirmed using ICC. In **Fig 7**, there was no noticeable difference in P2X4R expression between the ATP-exposed cells and the control group. The results aligned well with the findings reported by Toyomitsu *et al*. [39] that suggested that external stimuli increase P2X4R surface expression without affecting total levels of P2X4R (surface and intracellular) protein expression. As a result, the fluorescence intensity of P2X4R was unchanged in the control versus ATP-treated cells. Furthermore, the signal intensity for Iba1 (red) in 50 μM ATP treated cells was brighter compared to the control, suggesting Iba1 overexpression in ATP-treated cells. Similarly, an increase in the red fluorescence intensity suggested increased BDNF levels in ATP-treated cells. Taken together, ATP exposure increased intracellular Iba1 expression in SIM-A9 cells. Also, ATP-mediated activation of P2X4R increased intracellular BDNF levels in SIM-A9 microglia cells.

## Discussion

Our laboratory is developing LNP/siBDNF as a novel therapeutic approach to downregulate microglial BDNF and decrease pain hypersensitivity in a rat model of neuropathic pain. The goal of this study was to develop and characterize an activated *in vitro* microglia cell line model that can be utilized for screening LNP/siBDNF formulations. Lesions to the peripheral nervous system caused by mechanical trauma, metabolic disorders, neurotoxic chemicals, infection or tumor invasion lead to peripheral neuropathic pain. On the other hand, spinal cord injury, stroke, or multiple sclerosis-mediated damages to CNS also cause central neuropathic pain [40]. Numerous studies have demonstrated that PNI induces the release of ATP from spinal dorsal neurons that activates microglia in the spinal cord [13, 14, 21, 31]. ATP binds to P2X4R expressed in activated microglia, and stimulation of P2X4R activates the p-38 mitogen-activated protein kinase (MAPK) secondary messaging cascade leading to the synthesis and release of BDNF from microglia [14, 31]. The released BDNF acts as a microglia-neuronal signaling molecule by binding to tyrosine kinase B receptors (TrkB) on dorsal horn lamina I neurons and downregulates KCC2 causing disinhibition of neurons with a concomitant increase in chloride ion concentrations. The increase in [Cl^-^] is to an extent that GABA becomes excitatory and feeds forward towards NMDA potentiation [12, 13, 16, 19, 41]. Together these changes increase the ascending output of the dorsal horn and are sufficient for the maintenance of pain hypersensitivity, the key feature in neuropathic pain [12, 13, 16, 41].

We characterized the classical microglia marker, Iba1 [27, 42] and intracellular proteins involved in the ATP-stimulated P2X4R-BDNF signalling axis. We present three novel findings: *first*, SIM-A9 cells expressed P2X4R and BDNF proteins, *second*, ATP, but not LPS, was cytocompatible with SIM-A9 cells and *third*, exposure of cells to optimized ATP concentrations for defined periods increased the intracellular expression of Iba1 and BDNF proteins. Increased Iba1 levels confirmed activation of SIM-A9 microglia and increased BDNF expression confirmed ATP-mediated stimulation of the P2X4R- signalling pathway, a hallmark of chronic neuropathic pain pathophysiology [12, 31].

It has been previously reported that the SIM-A9 cell line, unlike virally-transformed microglia cell lines, showed features comparable to primary microglia and expressed microglial phenotypes such as CD68 or Iba1 [27]. Nagamoto-Combs *et al*. have demonstrated the microglia/macrophage-like properties of SIM-A9 cells and their phagocytic potential [27]. The authors have also demonstrated that the stimulation of SIM-A9 cells using inflammatory stimuli such as LPS or amyloid β_1–42_ increased the release of tumor necrosis factor-α and the production of nitric oxide [27]. In another study, Alhadidi and Shah investigated the role of the cytoskeletal protein, cofilin in LPS-mediated microglia activation utilizing the SIM-A9 cell line to study neuronal apoptosis during oxygen-glucose deprived conditions [43]. Incubation of cofilin siRNA in cell culture media for 72 h showed a statistically significant reduction in NF-κB and JAK-STAT-mediated cofilin expression, and the release of nitrogen oxide and TNF-α in LPS activated SIM-A9 cells [43]. As stated earlier, our primary objective was to determine if the SIM-A9 cells expressed P2X4 receptor and intracellular BDNF—and if ATP stimulation influences their expression levels. First, we characterized resting SIM-A9 cells for intracellular Iba1, P2X4R and BDNF proteins using ICC, western blot and In-cell western techniques. Though immunofluorescent staining is a widely used method to detect proteins of interest in cultured cells [44], known limitations associated with antibody specificity and selectivity result in false-positive and false-negative staining [45]. The above limitations urged us to use orthogonal methods to confirm protein expression. ICC allowed us to study cell morphology and confirm the intracellular expression of P2X4R, Iba1, and BDNF proteins in SIM-A9 cells. Despite providing a visual confirmation of protein expression, ICC did not allow us to distinguish specific isoforms, confirm the subcellular localization of proteins and eliminate the possibility of staining potential degradants or non-specific binding to other proteins that share common epitopes. Thus, we used western blotting of total cell lysates as another method to confirm the proteins of our interest.

Two sequential passages of SIM-A9 showed characteristic bands of α-tubulin (50 kD), P2X4R (43 kD) and Iba1 (17 kD). We observed the three different isomers of BDNF: pro-BDNF, BDNF dimer and monomer at 37, 28 and 14 kD, respectively (**Fig 2**). BDNF is intracellularly synthesized as a precursor pro-BDNF (37 kD) and subsequently cleaved by protease enzymes to form mature BDNF (BDNF, 14 kD) [46, 47]. Pro-BDNF acts not only as an intermediary of BDNF synthesis but also as an inflammatory mediator in inflammatory pain [47, 48]. Pro-BDNF interacts preferentially with the pan-neurotrophin receptor p75 (p75^NTR^), whereas BDNF selectively binds and activates the receptor tyrosine kinase TrkB, hence, reportedly showed opposite effects in terms of pro-BDNF/p75-mediated depression of GABA_A_R-synaptic activity and BDNF/TrkB-mediated induction of GABAnergic long-term potentiation [46, 47]. Thus, it is important to correlate the synthesis and expression of pro-BDNF and BDNF in normal and neuropathic pain conditions. In this present study, we focused on the expression of BDNF in microglia that is subsequently involved in BDNF-mediated downstream potentiation of pain hypersensitivity. There seemed to be a mild effect of passage number on the expression of Iba1 and BDNF dimer. Nagamoto-Comb *et al*. also observed a reduction in Iba1 signal intensity using ICC from SIM-A9 P7 to P40 [27]. The possible reason could be passage-dependent changes in microglia morphology and phenotypes. A previous study reported the decreased ability of SIM-A9 to express M2 phenotype markers such as COX-2 and Arg-1 after extended culturing, which may be associated with a gradual decrease in marker protein in immortalized cells [27].

Lastly, we optimized and established an ICW assay for detecting Iba1, P2X4R and BDNF in SIM-A9 microglia. We optimized the choice of primary and secondary antibodies, their dilution, different fixatives, their incubation time, and the effect of the presence of a permeabilizer in the blocking and antibody solutions (**S4 and S5 Figs**). ICW has multiple advantages over western blotting such as lower experimentation time, ease of high throughput screening, normalization mediated accuracy using multiple controls and total cell numbers, and allows qualitative and semi-quantitative expression of target proteins [49]. Confirmation of protein expression using the ICW technique can be influenced by at least three factors, including specificity of the primary antibody, the specificity of the fluorescent secondary antibody binding to the primary antibody, and fluorophore signal controls inferring that the observed fluorescence is due to the added secondary antibodies, not as a result of endogenous labeling of cellular components [50]. Hoffman *et al*. developed a high-throughput ICW method for mTORC1 signaling knockdown using siRNA and small molecule inhibitors demonstrating a statistically robust ICW assay for screening of therapeutics [51].

To establish an ICW assay for detecting the intracellular Iba1, BDNF, and surface/cytosolic P2X4R proteins, we evaluated the effect of two major components: fixatives and permeabilizer. In the absence of fixatives, we observed a complete cell loss **(S5B Fig)** due to multiple washing steps involved in the ICW protocol. Thus, we were required to include a fixative to detect P2X4R expression. We tested fixatives such as paraformaldehyde, a mixture of ethanol and glacial acidic acid (GAA), and ice-cold methanol. Furthermore, Triton X-100 was used as a permeabilizer to stain the intracellular proteins. The absence of a permeabilizer would have stained the surface receptors. In **S5A Fig**, the cells were fixed with either paraformaldehyde or ethanol/methanol/GAA in the absence of a permeabilizer. However, we could not detect surface P2X4R. The role of a fixative is to immobilize the cells by cross-linking with the cell membrane. It is known that the cross-linking and permeabilizing characteristics of fixatives [52, 53] might damage, degrade or denature the cell membrane and surface receptors. That could be the reason behind the lack of P2X4R expression in paraformaldehyde-fixed SIM-A9 cells without Triton-X100 (permeabilizer) (**S5A Fig)**. We speculate that surface detection of P2X4R requires live SIM-A9 cells and the use of a sensitive and quantitative assay such as flow cytometry [54]. P2X4R bands were observed in the groups when the cells were treated with a permeabilizer **(Fig 3** and **S5A Fig)** suggesting the cytosolic localization of P2X4R.

Makoto *et al*. explained the fate of cytosolic P2X4R in microglia [32]. After 3–7 days post-PNI, P2X4R levels increase in microglia residing in the spinal dorsal horn [32]. PNI increases fibronectins that upregulate P2X4R transcription and translation via phosphatidylinositol 3‐kinase (PI3K)–Akt, mitogen‐activated protein kinase (MAPK) and mitogen-activated protein kinase (MEK)–extracellular signal‐regulated kinase (ERK) signaling cascades in microglia. Intracellular P2X4R proteins are predominantly localized in lysosomes and remained stable against glycan degradation. P2X4Rs are a type of non-selective cation (Ca^+2^, Na^+,^ and K^+^ ions) channels, and upon ATP-binding, P2X4R opens the calcium channels allowing the rapid flow of calcium into microglia [20, 21, 32]. Calcium influx leads to exocytosis of the lysosomal P2X4R to the microglial cell membrane. In summary, ICC, western blot and ICW combinedly confirmed Iba1, P2X4R and BDNF expression in SIM-A9 cells. Technical limitations associated with the use of a fixative in ICC/ICW studies precluded the surface detection of P2X4R and our future studies will use a more sensitive flow cytometry assay [54] to detect surface receptors.

Prior to evaluating the effect of stimulators on the microglia phenotype, we optimized experimental parameters to determine their safe concentration, incubation time, and post-treatment cytocompatibility. We investigated the cytocompatibility of LPS, the most widely used inflammatory stimulator for activating microglia *in vitro* [26, 27, 33], and ATP, a P2X4R-selective stimulator [31, 55]. Cytocompatibility of LPS and ATP was evaluated using two different assays, ATP and MTS assay. The ATP Assay **(Fig 4)** was used as a predominant method due to the rapid and sensitive nature of the assay for evaluating the cell viability of LPS- and ATP-treated cells [56, 57]. MTS assay **(S6 Fig)** was used as an orthogonal method to confirm the cell viability and to avoid any possible influence of ATP treatment on the ATP assay readout. Nagamoto-Combs *et al*. studied the effect of 2.5 ng/mL LPS-mediated synthesis of nitric oxide and the release of TNF-α from SIM-A9 cells [27], but the effect of LPS treatment on cell viability was not reported. Therefore, we investigated LPS cytocompatibility with SIM-A9 cells at concentrations ranging from 2.5 ng/mL to 50 μg/mL. SIM-A9 showed no effects of LPS toxicity when the cell viability was measured immediately after a 4 h incubation. However, cells incubated with LPS at a concentration as low as 2.5 ng/mL for 24 h showed a significant reduction in SIM-A9 cell viability. Severe toxicity was observed at 24 and 48 h post-LPS treatment. Notably, the cytocompatibility studies for LPS and ATP were performed using a serum-containing treatment medium. We performed an additional experiment to determine the effect of serum on cell viability. Cells were exposed to 4 h LPS in serum-free (w/-) and serum-containing (w/+) culture conditions. Interestingly, there was no significant difference in 48 h post-treatment cell viability among groups that were exposed to LPS either in the absence or presence of serum **(S16 Fig)**. On the other hand, ATP was well tolerated by SIM-A9 cells incubated for 24 h even at a high concentration of 250 μM. No toxicity was observed immediately, 24 and 48 h post-ATP treatment. Importantly, the favorable cytocompatibility of ATP with SIM-A9 cells is an encouraging finding that supports our efforts to develop neuropathic pain therapeutics targeting the ATP-P2X4R-BDNF signaling pathway. Therefore, SIM-A9 cells were exposed to ATP in all subsequent experiments.

A low, medium and high ATP concentration i.e. 1, 50 and 100 μM were used to stimulate SIM-A9 cells and determine the stimulation-induced changes in protein expression. Fifty μM ATP stimulation did not alter the morphology of SIM-A9 cells in ICC microscopic images, however, a considerable increase in Iba1 and BDNF signals in each cell were observed compared to control, untreated cells (**Fig 7**). Western blot analysis of ATP-stimulated cells showed no significant changes in P2X4R levels. We did not expect to see any ATP-mediated changes in total P2X4R protein (surface and intracellular) expression since ATP exposure only trafficks P2X4R to the cell surface without affecting the total expression levels of P2X4R proteins [39]. Consequently, it increases the expression of BDNF that leads to the overall activation of microglia. Moreover, our studies consistently showed that cells stimulated with 50 μM ATP for 2–4 h showed maximum Iba1, BDNF (14 kD) and BDNF dimer (28 kD) compared to other tested ATP concentrations and incubation times. Noteworthy, the results of our studies were in agreement with the results reported from studies performed in primary microglia cultures by Trang *et al*. [31]. However, we did not observe the biphasic overexpression of BDNF that was reported in their studies using primary microglia. One of the limitations of the current study lies in the fact that we have not directly established that the ATP-mediated activation proceeds via the P2X4R pathway. This could be done by selectively silencing P2X4R expression in these cells either at the genetic level or protein expression level and then determining its effects on BDNF and Iba1 levels. We wish to reiterate that the goal of this study is to determine if the SIM-A9 cell line expresses high amounts of BDNF to conveniently screen LNP/siBDNF formulations in a fast-growing cell line. This will allow us to optimize the transfection parameters such as LNP composition, siRNA sequences, dose, transfection time, etc. Our future studies will use primary microglia to run the siBDNF transfection experiments once we optimize the above parameters. We will establish the mechanistic axis in primary microglia as they are more representative of cells *in vivo*.

Overall, the use of optimized parameters and assay protocols reported herein enables robust and reproducible activation of SIM-A9 microglia *in vitro*. Our current studies are focussed on testing BDNF knockdown using BDNF siRNA delivered using lipidoid nanoparticles in SIM-A9 microglia as a novel chronic neuropathic pain therapy.

## Conclusion

SIM-A9 expressed microglial phenotypes such as P2X4R, Iba1, and BDNF that are involved in the pathogenesis of PNI-induced neuropathic pain. ATP at a safe dose showed a time-dependent increase in Iba1 and BDNF expression without causing intracellular toxicity. LPS stimulation was found to be toxic to SIM-A9 cells. Hence, an ATP activated SIM-A9 cell line model system can be utilized for screening of neuropathic pain therapeutics targeting P2X4R and/or BDNF knockdown, including small- as well as macro-molecular therapies such as proteins and nucleic acids.

## Supporting information

S1 Fig Representative ICC image of control SIM-A9 cells showing P2X4R expression acquired under brightfield settings. Scale bar: 100μm. The larger image is the raw object captured using the EVOS microscope and the smaller image is the cropped image demonstrated in **the main text in Figs 1B and 7**.
(DOCX)

S2 Fig Alpha-tubulin, BDNF, P2X4R, and Iba1 expression in the U-87MG and SIM-A9 cell lines. **A)** U-87MG cell lines at P131 and P132 were cultured in complete growth medium for 72 h in T-75 flasks. The cells were dissociated using TrypLE express and were lysed in Tris-HCl pH 8.0: 4% SDS at 1:1 ratio containing 10 μg/mL aprotinin. Total protein concentration was calculated using BCA assay, and the lysates were loaded at 40 and 50 μg/lane. α-tubulin (50 kD), BDNF (14 kD) and its isomers (28 and 37 kD) were expressed in U-87 MG cells. **B)** SIM-A9 at P4 and P5, and U-87MG at P131 and P132 were cultured, lysed, and cell lysates were loaded in an SDS-PAGE gel at 30, 40 and 50 μg/lane. Iba1 (17 kD) was expressed in SIM-A9 and U-87MG cell lysates. P2X4R (43 kD) was expressed in SIM-A9, but not in the U-87MG cell line. The white dotted squares from raw blots **A** and **B** were shown in **S2 Fig**. The order of loading the protein ladder and experimental samples were the same in raw blots A and B and **S2A and S2B Fig**, respectively.
(DOCX)

S3 Fig Raw western blots for Fig 2 in the main text. The white dotted squares from raw blots **A** and **B** were shown in **Fig 2**. The order of loading the protein ladder and experimental samples were the same in raw blots A and B and **S3A and S3B Fig**, respectively.
(DOCX)

S4 Fig ICW parameter optimization for Iba1 detection in SIM-A9 cells. SIM-A9 cells were fixed with 4% PFA for 20 min. Non-specific binding of antibodies was blocked using a Li-COR Odyssey blocking buffer. Cells were immunostained using rabbit primary antibodies against Iba1 as indicated. Cells were then stained with goat or donkey anti-rabbit AF790 at a 1:700 (red dotted areas) or a 1:8000 dilution (yellow dotted areas). The plate was scanned using an Odyssey imager at intensity setting 5, plate height 4.0 mm and processed using ImageStudio 5.2 software. The goat anti-rabbit secondary antibody at 1:700 dilution showed intense fluorescence with low background. Secondary antibodies at 1:8000 dilution showed reduced fluorescence signals. Anti-rabbit secondary antibodies exhibited lower fluorescence signals compared to the goat species. The images presented are representative of two independent experiments with triplicate wells per group. Images A-C are raw ICW images obtained from the Odyssey imager at 700 nm (red) and 800 nm (green) channels. The white dotted square in images **A-C** was presented in **the main text in Fig 3**, whereas the yellow dotted square in image **A** is presented in **S4 Fig**.
(DOCX)

S5 Fig A) ICW parameter optimization for P2X4R detection in SIM-A9 cells fixed with varying concentrations of the fixatives. B) ICW without fixatives for SIM-A9 cells at different ATP and LPS treatment conditions. A) SIM-A9 cells were fixed using either 1%, 2% or 4% PFA for 10 or 20 min. Selected wells were also fixed with 95% ethanol and 5% glacial acetic acid mixture or ice-cold methanol for 10 min. In addition to studying the effect of various permeabilizing agents, we also used intact or lysed cells (w/o or treated w- Triton X-100). Non-specific binding of antibodies was blocked using a blocking buffer. Cells were immunostained with mouse primary antibodies against P2X4R (1:250 dilution) as indicated. Cells were then stained with donkey anti-mouse AF790 at 1:700. B) SIM-A9 cells were cultured for 48 h and treated with different concentrations of ATP/LPS for 2 and 4 h. The cells were not fixed. Cells were blocked using a blocking solution and incubated with primary and secondary antibodies. The plate was scanned using Odyssey imager at intensity setting 5, plate height 4.0 mm and processed using ImageStudio 5.2 software. The images presented are representative of two independent experiments with triplicate wells per group. The raw blots for S5A and S5B Fig were shown as Raw blot for A and B respectively.
(DOCX)

S6 Fig Cytocompatibility of LPS and ATP with SIM-A9 cells determined using MTS assay. **A**) Experiment scheme: SIM-A9 cells were cultured for 48 h and exposed to 2.5 to 25000 ng/mL LPS for 4 or 24 h. Cells were incubated with 25 nM to 250 μM ATP for 4 or 24 h. Cell Titer Glo ATP assay was performed immediately after exposure (**B and E**), 24 h (**C and F**), and 48 h post-ATP exposure (**D and G**). The viability of LPS/ATP treated cells was calculated relative to the control, untreated cells. Statistical analysis was performed using GraphPad Prism 8.1.2. Asterisks indicate significant differences (**** p<0.0001, *** p<0.001, ** p<0.005, * p<0.05) compared to the control. The data is representative of two independent experiments and is presented as mean ± standard deviation (SD) of at least n = 4 wells per group.
(DOCX)

S7 Fig Effect of 4 h LPS exposure on SIM-A9 cells observed using trypan blue assay. Cells were incubated with LPS at different concentrations for 4 h in serum-containing treatment medium. After 48 h, the cells were dissociated from the plate and added into microcentrifuge tubes. Trypan blue dye was added at a 1:1 v/v ratio to the cell suspension and incubated for 5–10 min. Ten μL of the mixture was pipetted on a slide that was then were inserted in the Auto Cell counter (CountessII).
(DOCX)

S8 Fig Effect of 24 h LPS exposure on SIM-A9 cells observed using trypan blue assay. Cells were incubated with LPS at different concentrations for 24 h in serum-containing treatment medium. After 48 h, the cells were dissociated from the plate and added into microcentrifuge tubes. Trypan blue dye was added at a 1:1 v/v ratio to the cell suspension and incubated for 5–10 min. Ten μL of the mixture was pipetted on a slide that was then were inserted in the Auto Cell counter (CountessII).
(DOCX)

S9 Fig Microscopic images of control and LPS treated SIM-A9 in a 96-well plate. SIM-A9 cells were cultured for 48 h and exposed to 2.5 to 25,000 ng/mL LPS for 24 h. The treatment medium was replaced with the complete growth medium and incubated for 48 h. The images were acquired using an EVOS microscope at 10x magnification. The scale bar is 400 μm.
(DOCX)

S10 Fig Microscopic images of control and LPS treated SIM-A9 cells in a 96-well plate. Cells were first exposed to LPS at the indicated concentrations for 24 h. The cells were then imaged 48 h-post exposure to LPS. The images were acquired using an EVOS microscope at 4x magnification. The scale bar is 1000 μm.
(DOCX)

S11 Fig Effect of 50 μM ATP stimulation on SIM-A9 cells in serum-containing or serum-free treatment media determined using western blotting (A). Densitometry analysis of BDNF (B), BDNF dimer (C), and Iba1 (D) was performed using Image Studio 5.2. Normalized signal intensity in Y-axis represents the normalization of protein signal intensity to, first, α-tubulin signal intensity followed by normalization to the signal intensity of control, untreated cells. The order of loading the protein ladder and experimental samples were the same in the raw blot and S11A.
(DOCX)

S12 Fig Densitometry analysis for BDNF and Iba1 of three independent western blot experiments demonstrating ATP-mediated modulation in expression levels of BDNF and Iba1. Normalized signal intensity in Y-axis represents a normalization of protein signal intensity to, first, α-tubulin signal intensity followed by normalization to the signal intensity of control/untreated cells at each time point. Data are presented as mean ± SD of n = 3 samples.
(DOCX)

S13 Fig Raw western blots for Fig 5 in the main text. The sign “X” indicates that the part of the blot was not depicted in **Fig 5**.
(DOCX)

S14 Fig Raw images of ICW obtained using an odyssey imager at 700 nm (red) and 800 nm (green) channels. The white squares in images were presented in **Fig 6A in the main text** demonstrating Iba1 expression, whereas red dotted squares in these images were depicted in **Fig 6C in the main text** demonstrating BDNF expression.
(DOCX)

S15 Fig ICW of BDNF expression in 4h LPS treated SIM-A9 cells. A) SIM-A9 cells were cultured for 48 h in a 96-well plate. The cells were treated with 2.5 and 100 ng/mL LPS for 4 h in complete growth media. Immediately after LPS treatment, cells were fixed, washed, blocked, and stained for cell nuclei (DAPI, red) and BDNF (green). B) Densitometry analysis was performed using Image Studio 5.2. Normalized BDNF signal intensity in the Y-axis represents the normalization of green signal intensity to DAPI signal intensity followed by subtraction of non-specific secondary staining.
(DOCX)

S16 Fig Cell viability of SIM-A9 48 h-post LPS treatment in serum-containing and serum-free medium determined using MTS assay. SIM-A9 cells were cultured for 48 h and exposed to 2.5 to 25000 ng/mL LPS for 4 h. MTS assay was performed 48 h post-LPS exposure. The viability of LPS-treated cells was calculated relative to the control group. Statistical analysis was performed using GraphPad Prism 8.1.2. Asterisks indicate significant differences (**** p<0.0001, *** p<0.001, ** p<0.005, * p<0.05) compared to the control. The data is representative of two independent experiments and is presented as mean ± standard deviation (SD) of at least n = 4 wells per group.
(DOCX)

S1 Table Densitometry analysis of P2X4R band densities showing the effect of ATP stimulation at the 2h time point. (DOCX)

## Abbreviations

ATP: adenosine triphosphate
BCA: Bicinchoninic acid
BDNF: brain-derived neurotrophic factor
ICW: In-Cell Western
ICC: immunocytochemistry
Iba1: ionized calcium-binding adapter molecule
LPS: lipopolysaccharide
MTS: 3-(4,5-dimethylthiazol-2-yl)-5-(3-carboxymethoxyphenyl)-2-(4-sulfophenyl)-2H-tetrazolium
P2X4R: P2X4 purinoceptors
SIM-A9: spontaneously immortalized microglia
