# Supplementary material for: Characterization of the SIM-A9 cell line as a model of activated microglia in the context of neuropathic pain
Source: PLoS One. 2020 Apr 14;15(4):e0231597. doi: 10.1371/journal.pone.0231597 (PMC7156095; doi:10.1371/journal.pone.0231597)
Supplement: S1 Fig — Scale bar: 100μm. The larger image is the raw object captured using the EVOS microscope and the smaller image is the cropped image demonstrated in the main text in Figs 1B and 7. (DOCX) [file pone.0231597.s001.docx]

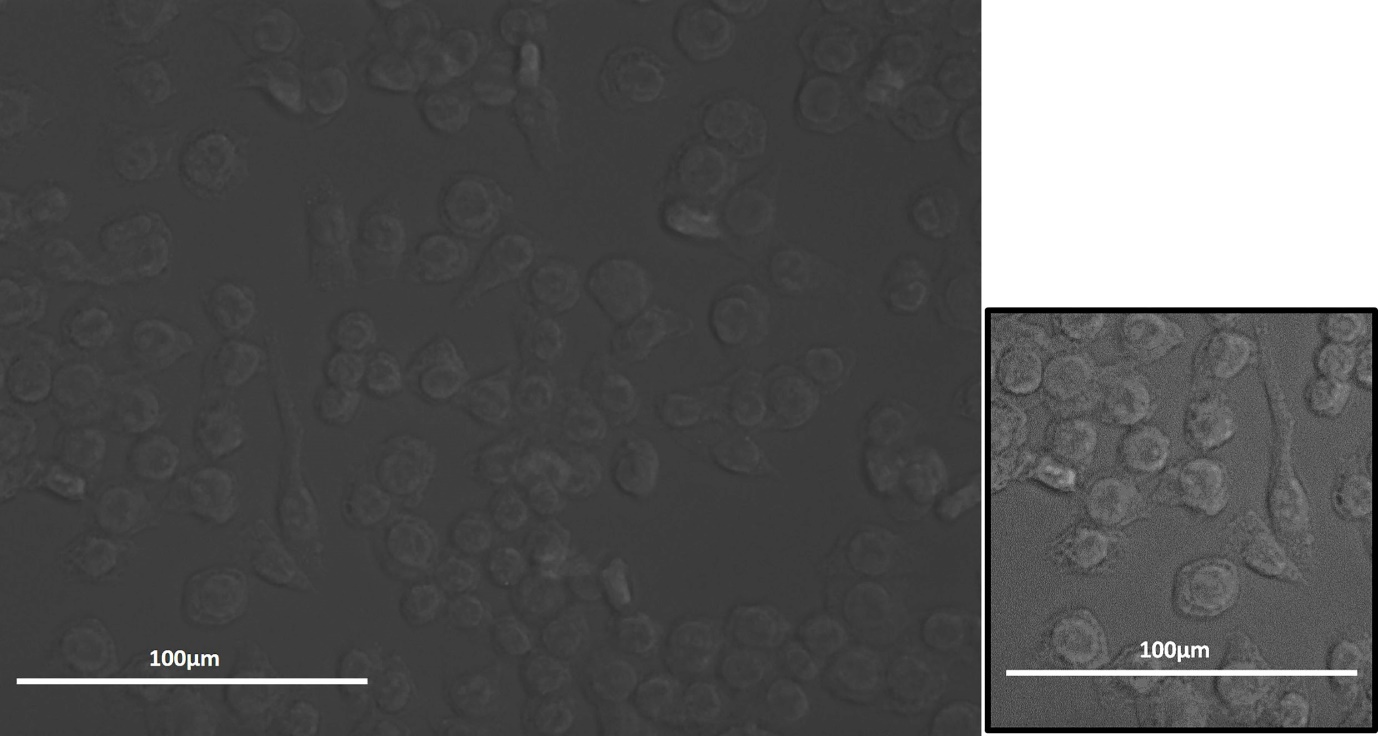


**S1 Fig.** **Representative ICC image of control SIM-A9 cells for P2X4R expression under brightfield settings imaged using EVOS FL (Invitrogen, Bothell, WA).** Scale bar: 100µm. The larger image is the raw object captured using the EVOS microscope and the smaller image is the cropped image demonstrated in **the main text in Figs 1B and 7**.
