# Supplementary material for: Characterization of the SIM-A9 cell line as a model of activated microglia in the context of neuropathic pain
Source: PLoS One. 2020 Apr 14;15(4):e0231597. doi: 10.1371/journal.pone.0231597 (PMC7156095; doi:10.1371/journal.pone.0231597)
Supplement: S2 Fig — A) U-87MG cell lines at P131 and P132 were cultured in complete growth medium for 72 h in T-75 flasks. The cells were dissociated using TrypLE express and were lysed in Tris-HCl pH 8.0: 4% SDS at 1:1 ratio containing 10 μg/mL aprotinin. Total protein concentration was calculated using BCA assay, and the lysates were loaded at 40 and 50 μg/lane. α-tubulin (50 kD), BDNF (14 kD) and its isomers (28 and 37 kD) were expressed in U-87 MG cells. B) SIM-A9 at P4 and P5, and U-87MG at P131 and P132 were cultured, lysed, and cell lysates were loaded in an SDS-PAGE gel at 30, 40 and 50 μg/lane. Iba1 (17 kD) was expressed in SIM-A9 and U-87MG cell lysates. P2X4R (43 kD) was expressed in SIM-A9, but not in the U-87MG cell line. The white dotted squares from raw blots A and B were shown in S2 Fig. The order of loading the protein ladder and experimental samples were the same in raw blots A and B and S2A and S2B Fig, respectively. (DOCX) [file pone.0231597.s002.docx]

*
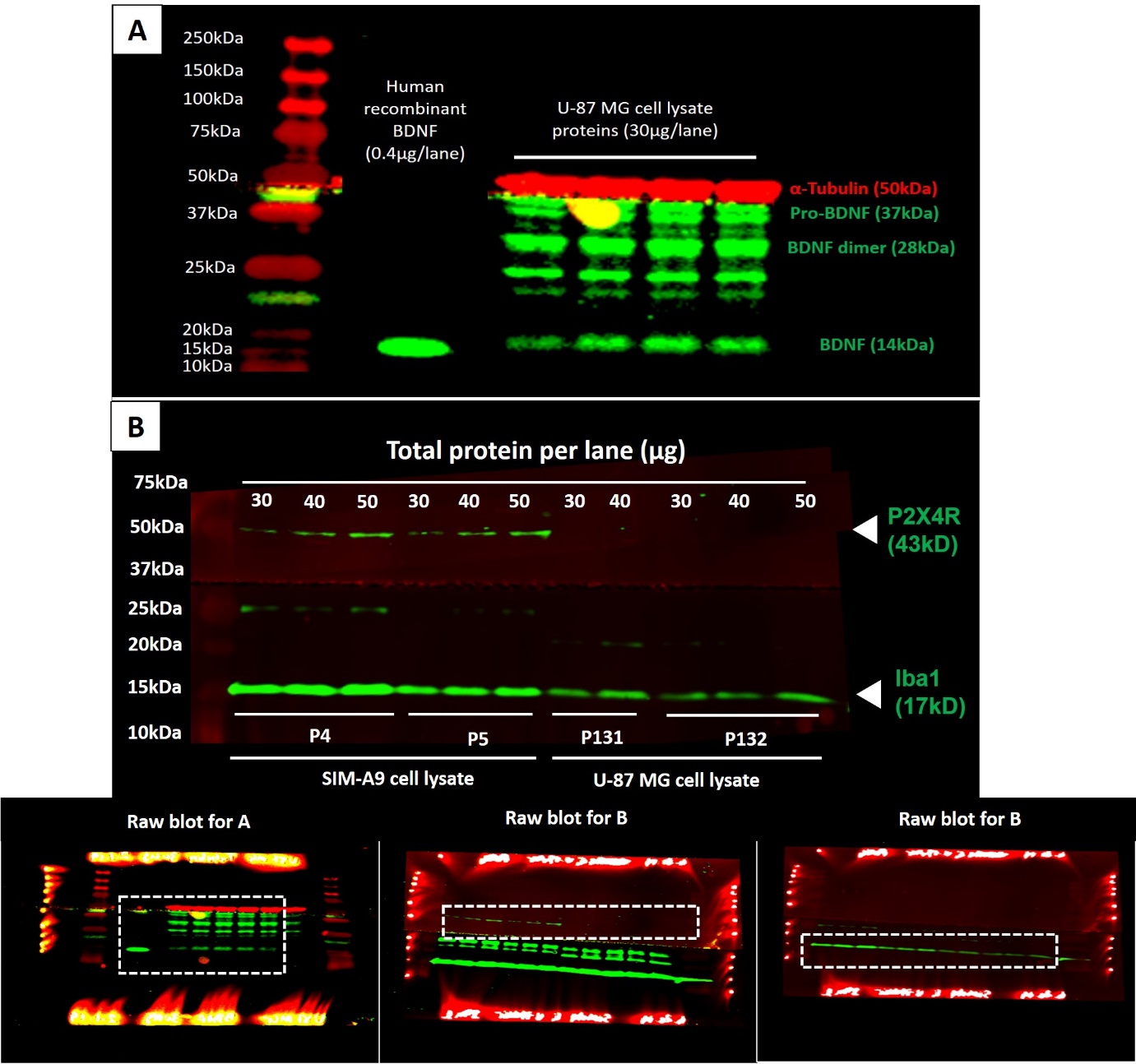
*

**S2 Fig**: **α-tubulin, BDNF, P2X4R, and Iba1expression in the U-87 and SIM-A9 cell lines.** **A)**U-87 MG cell lines at P131 and P132 were cultured in complete growth medium for 72 h in T-75 flasks. The cells were dissociated using TrypLE express and were lysed in Tris HCl pH 8.0: 4%SDS at 1:1 ratio containing 10µg/mL aprotinin. Total protein concentration was calculated using BCA assay, and the lysates were loaded at 40 and 50 µg/lane. α-tubulin (50kD), BDNF (14 kD) and its isomers (28 and 37 kD) were expressed in U-87 MG cells. **B)** SIM-A9 at P4 and P5, and U-87MG at P131 and P132 were cultured, lysed, and cell lysates were loaded in an SDS-PAGE gel at 30, 40 and 50 µg/lane. Iba1(17 kD) was expressed in SIM-A9 and U-87MG cell lysates. P2X4R (43 kD) was expressed in SIM-A9, but not in the U-87MG cell line. The white dotted squares from raw blots **A** and **B** were shown in **S2** **Fig**. The order of loading the protein ladder and experimental samples were the same in raw blots A and B and **S2A and B Figs**, respectively.
