# Supplementary material for: Characterization of the SIM-A9 cell line as a model of activated microglia in the context of neuropathic pain
Source: PLoS One. 2020 Apr 14;15(4):e0231597. doi: 10.1371/journal.pone.0231597 (PMC7156095; doi:10.1371/journal.pone.0231597)
Supplement: S3 Fig — The white dotted squares from raw blots A and B were shown in Fig 2. The order of loading the protein ladder and experimental samples were the same in raw blots A and B and S3A and S3B Fig, respectively. (DOCX) [file pone.0231597.s003.docx]

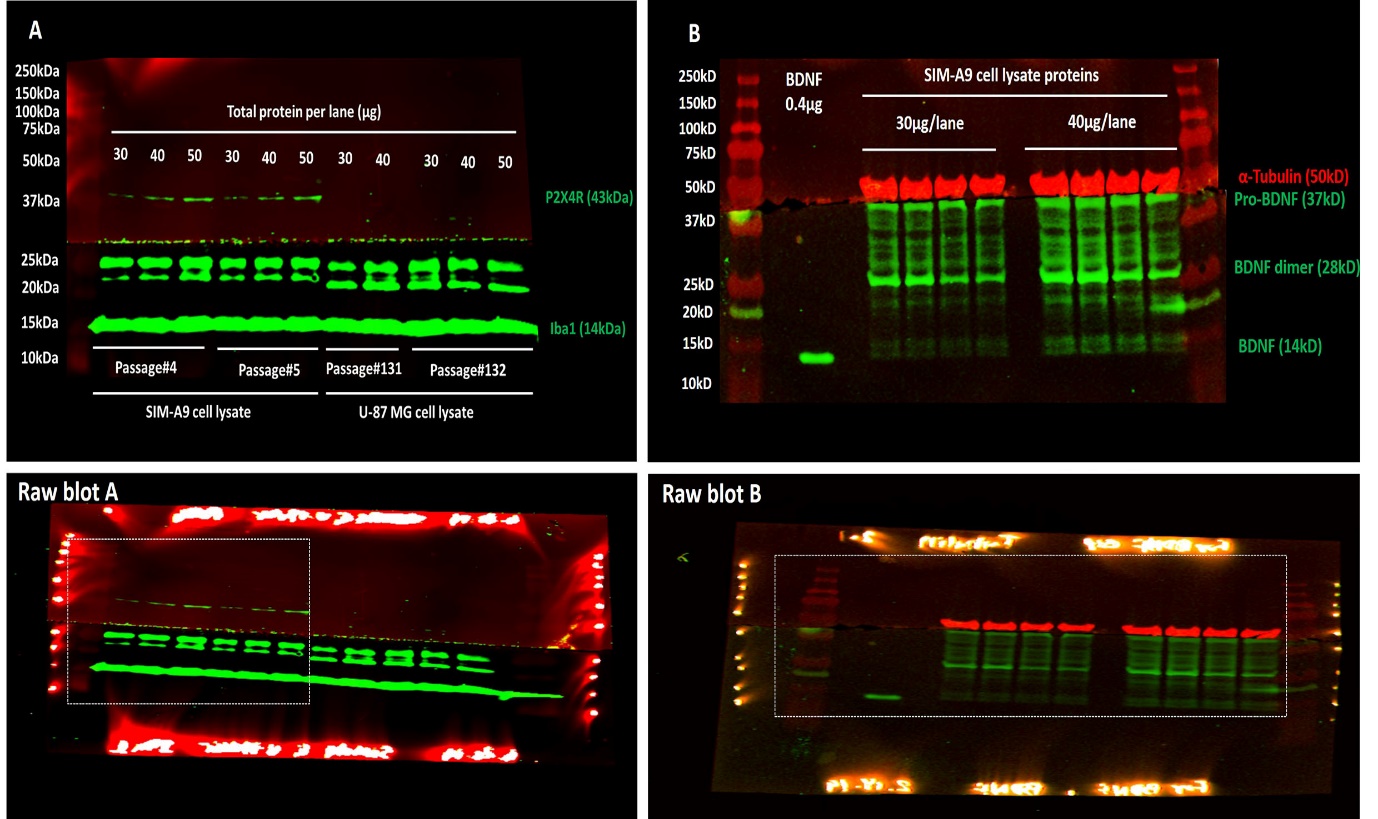


**S3 Fig.** Raw western blots for **Fig 2** in the main text. The white dotted squares from raw blots **A** and **B** were shown in **Fig 2**. The order and labeling of molecular weight markers in both the sides of **Raw blots A and B** are the same as **S3A** and B **Fig respectively** The order of loading the protein ladder and experimental samples were the same in raw blots A and B and **S3A and B Figs**, respectively.
