# Supplementary material for: Characterization of the SIM-A9 cell line as a model of activated microglia in the context of neuropathic pain
Source: PLoS One. 2020 Apr 14;15(4):e0231597. doi: 10.1371/journal.pone.0231597 (PMC7156095; doi:10.1371/journal.pone.0231597)
Supplement: S4 Fig — SIM-A9 cells were fixed with 4% PFA for 20 min. Non-specific binding of antibodies was blocked using a Li-COR Odyssey blocking buffer. Cells were immunostained using rabbit primary antibodies against Iba1 as indicated. Cells were then stained with goat or donkey anti-rabbit AF790 at a 1:700 (red dotted areas) or a 1:8000 dilution (yellow dotted areas). The plate was scanned using an Odyssey imager at intensity setting 5, plate height 4.0 mm and processed using ImageStudio 5.2 software. The goat anti-rabbit secondary antibody at 1:700 dilution showed intense fluorescence with low background. Secondary antibodies at 1:8000 dilution showed reduced fluorescence signals. Anti-rabbit secondary antibodies exhibited lower fluorescence signals compared to the goat species. The images presented are representative of two independent experiments with triplicate wells per group. Images A-C are raw ICW images obtained from the Odyssey imager at 700 nm (red) and 800 nm (green) channels. The white dotted square in images A-C was presented in the main text in Fig 3, whereas the yellow dotted square in image A is presented in S4 Fig. (DOCX) [file pone.0231597.s004.docx]

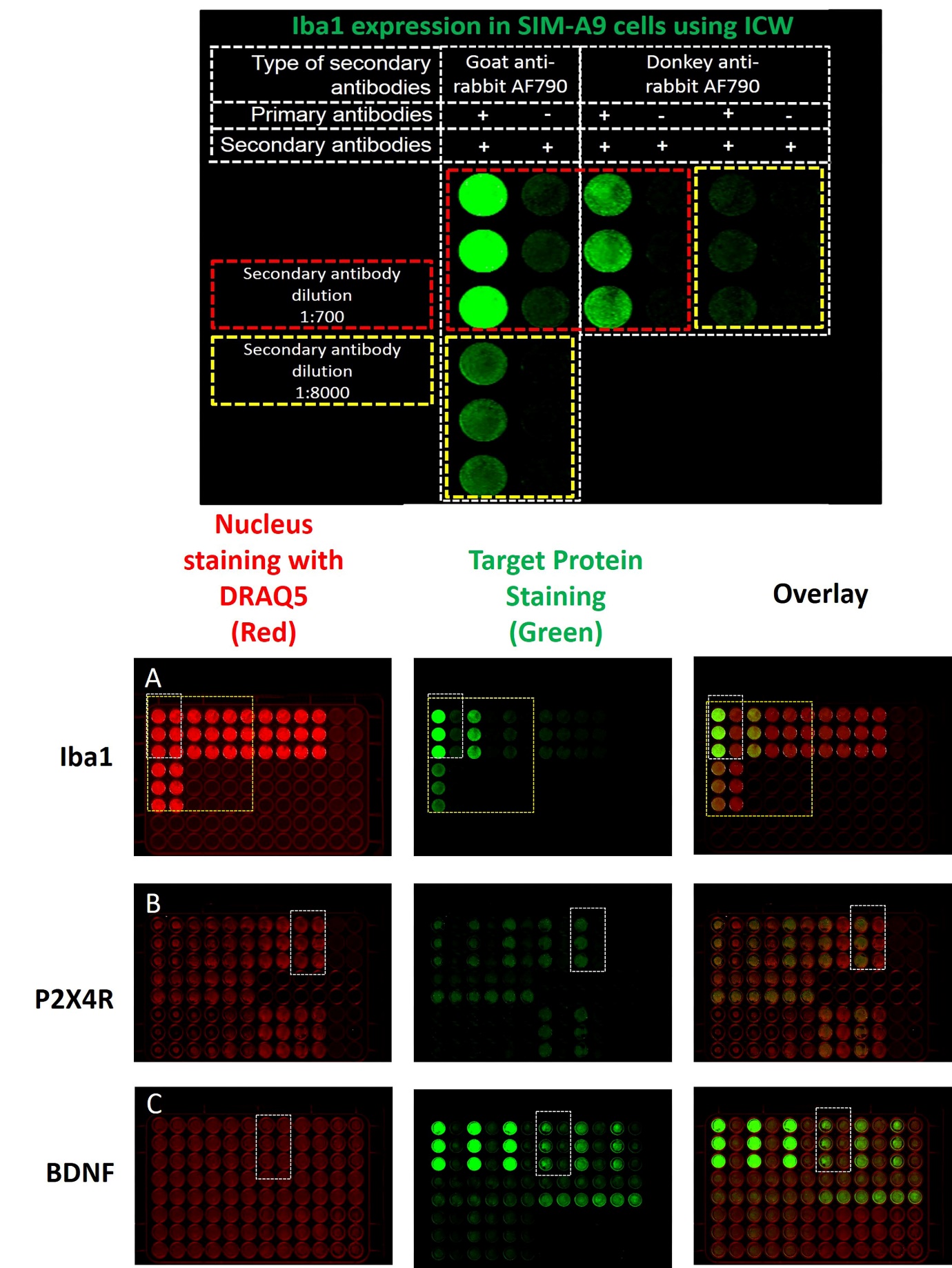


**S4 Fig.** **ICW parameter optimization for Iba1 detection in SIM-A9 cells.** SIM-A9 cells were fixed with 4% PFA for 20 min. Non-specific binding of antibodies was blocked using a Li-COR Odyssey blocking buffer. Cells were immunostained using rabbit primary antibodies against Iba1 as indicated. Cells were then stained with goat or donkey anti-rabbit AF790 at a 1:700 (red dotted areas) or a 1:8000 dilution (yellow dotted areas). The plate was scanned using an Odyssey imager at intensity setting 5, plate height 4.0 mm and processed using ImageStudio 5.2 software. The goat anti-rabbit secondary antibody at 1:700 dilution showed intense fluorescence with low background. Secondary antibodies at 1:8000 dilution showed reduced fluorescence signals. Anti-rabbit secondary antibodies exhibited lower fluorescence signals compared to the goat species. The images presented are representative of two independent experiments with triplicate wells per group. Images A-C are raw ICW images obtained from the Odyssey imager at 700nm (red) and 800nm (green) channels. The white dotted square in images **A-C** was presented in **the main text in Fig 3**, whereas the yellow dotted square in image **A** is presented in **S4 Fig.**

**Figure Discussion:** We optimized two parameters to detect intracellular Iba1 proteins using ICW: (1) Species of the secondary antibody, and (2) secondary antibody dilution. SIM-A9 cells, fixed with 4% PFA for 20 min, were immunostained using rabbit primary antibodies against Iba1. Cells were then stained with **goat or donkey** anti-rabbit AF790 at a **1:700** (red dotted areas) or a **1:8000** dilution (yellow dotted areas). The goat anti-rabbit secondary antibody at 1:700 dilution showed intense fluorescence with low background. Secondary antibodies at 1:8000 dilution showed reduced fluorescence signals. Anti-rabbit secondary antibodies exhibited lower fluorescence signals compared to the goat species.
