# Supplementary material for: Characterization of the SIM-A9 cell line as a model of activated microglia in the context of neuropathic pain
Source: PLoS One. 2020 Apr 14;15(4):e0231597. doi: 10.1371/journal.pone.0231597 (PMC7156095; doi:10.1371/journal.pone.0231597)
Supplement: S5 Fig — A) ICW parameter optimization for P2X4R detection in SIM-A9 cells fixed with varying concentrations of the fixatives. B) ICW without fixatives for SIM-A9 cells at different ATP and LPS treatment conditions. A) SIM-A9 cells were fixed using either 1%, 2% or 4% PFA for 10 or 20 min. Selected wells were also fixed with 95% ethanol and 5% glacial acetic acid mixture or ice-cold methanol for 10 min. In addition to studying the effect of various permeabilizing agents, we also used intact or lysed cells (w/o or treated w- Triton X-100). Non-specific binding of antibodies was blocked using a blocking buffer. Cells were immunostained with mouse primary antibodies against P2X4R (1:250 dilution) as indicated. Cells were then stained with donkey anti-mouse AF790 at 1:700. B) SIM-A9 cells were cultured for 48 h and treated with different concentrations of ATP/LPS for 2 and 4 h. The cells were not fixed. Cells were blocked using a blocking solution and incubated with primary and secondary antibodies. The plate was scanned using Odyssey imager at intensity setting 5, plate height 4.0 mm and processed using ImageStudio 5.2 software. The images presented are representative of two independent experiments with triplicate wells per group. The raw blots for S5A and S5B Fig were shown as Raw blot for A and B respectively. (DOCX) [file pone.0231597.s005.docx]

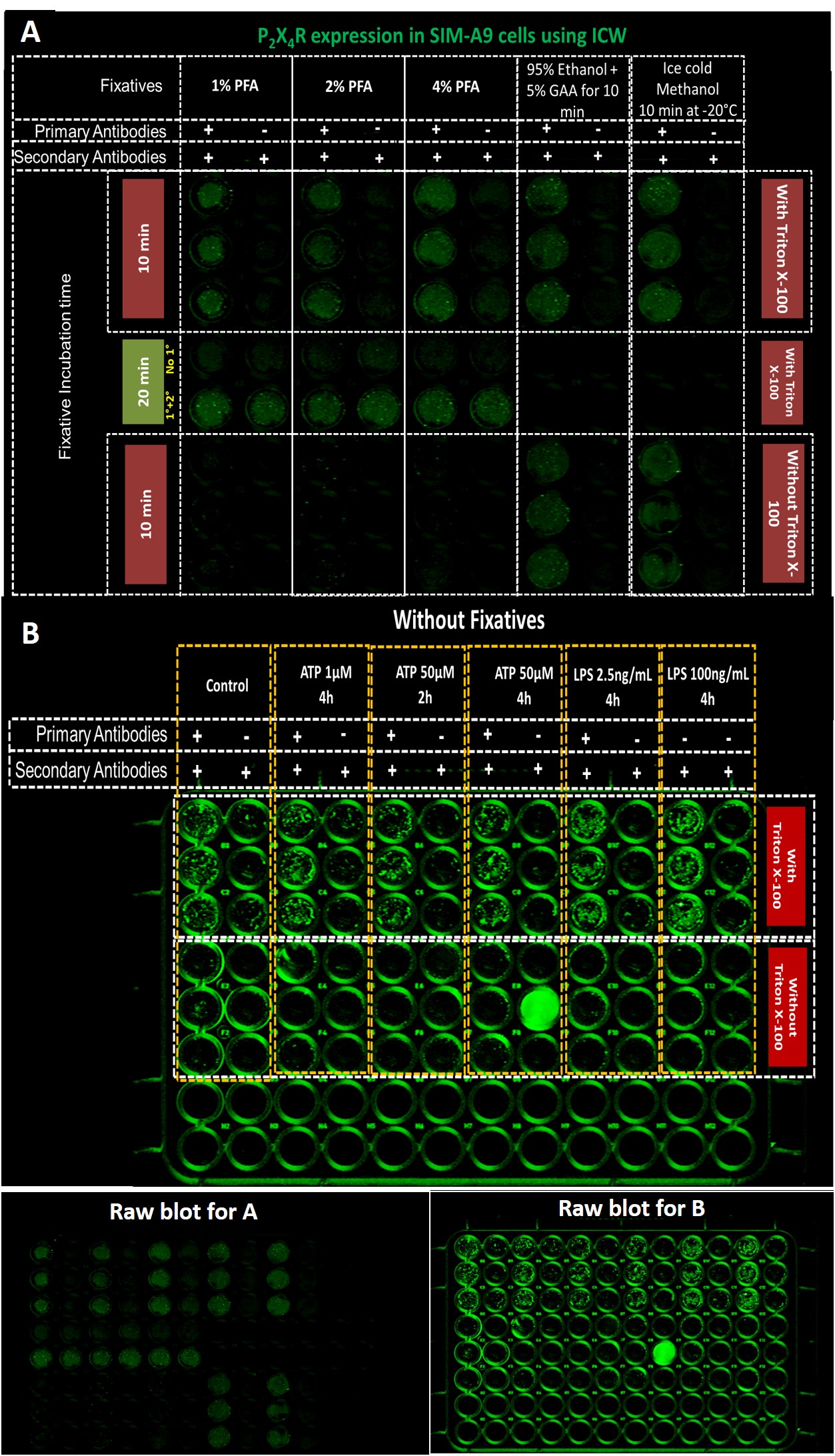


**S5 Fig.** **A) ICW parameter optimization for P2X4R detection in SIM-A9 cells fixed with varying concentrations of the fixatives. B) ICW without fixatives for SIM-A9 cells at different ATP and LPS treatment conditions.** **A)** SIM-A9 cells were fixed using either 1%, 2% or 4% PFA for 10 or 20 min. Selected wells were also fixed with 95% ethanol and 5% glacial acetic acid mixture or ice-cold methanol for 10 min. In addition to studying the effect of various permeabilizing agents, we also used intact or lysed cells (w/o or treated w- Triton X-100). Non-specific binding of antibodies was blocked using blocking buffer. Cells were immunostained with mouse primary antibodies against P2X4R (1:250 dilution) as indicated. Cells were then stained with donkey anti-mouse AF790 at 1:700. **B)** SIM-A9 cells were cultured for 48h and treated with different concentrations of ATP/LPS for 2 and 4 h. The cells were not fixed. Cells were blocked using a blocking solution and incubated with primary and secondary antibodies. The plate was scanned using Odyssey imager at intensity setting 5, plate height 4.0 mm and processed using ImageStudio 5.2 software. The images presented are representative of two independent experiments with triplicate wells per group. The raw blots for **S5A and B Figs** were shown as **Raw blot for A** and **B** respectively.

**Figure discussion:**

**Effect of Fixative agents and their incubation time (for P2X4R detection):** To detect surface and/or cytosolic P2X4R, we evaluated the effect of fixatives and its incubation time in ICW. A fixative agent is required for immobilizing cells both in ICW and ICC studies. The lack of a fixative in the protocol washed off the cells mainly after the addition of a blocking buffer (**S5B Fig**). Next, SIM-A9 cells were fixed using either 1%, 2% or 4% PFA for 10 or 20 min (**S5A Fig**). Selected wells were also fixed with 95% ethanol and 5% glacial acetic acid mixture or ice-cold methanol for 10 min. Cells were immunostained with mouse primary antibodies against P2X4R (1:250 dilution) as indicated. Cells were then stained with donkey anti-mouse AF790 at 1:700. Fixation with 4% PFA for 20 min showed higher signals compared to a lower PFA concentration and incubation time. The possible reason for low signals at lower PFA concentration and time could be linked to cell loss (as a result of the required multiple washing steps) due to inadequate fixative concentration and incubation time. It is known that the cross-linking and permeabilizing characteristics of fixatives [8, 9] might damage, degrade or denature the cell membrane and surface receptors. That could be the reason behind the lack of surface P2X4R expression in paraformaldehyde-fixed SIM-A9 cells (**S5A Fig)**.

**Effect of cell permeabilizer in antibody solution (for P2X4R detection):** Intracellular P2X4R proteins are predominantly localized in lysosomes, while ATP-mediated stimulation of microglia leads to calcium influx-mediated exocytosis of the lysosomal P2X4R to the microglial cell membrane. We tried to detect surface and intracellular P2X4R in the absence and presence of a cell permeabilizer (Triton X-100) in antibody solutions. SIM-A9 cells were fixed with different types of fixatives at varying concentrations. Cells were incubated with primary and secondary antibodies either with or without Triton X-100 in the antibody solutions. Interestingly, PFA fixation without Triton X-100 did not show any signals suggesting that Triton X-100 (permeabilizer) was required for detecting P2X4R using ICW. Besides, 95% ethanol and 5% glacial acetic acid (GAA) mixture and ice-cold absolute methanol showed P2X4R expression both with and without 0.3%v/v Triton X-100. The results suggested that ethanol/GAA or methanol acted as a fixative cum permeabilizer in this setup. Therefore, we used a 4% PFA fixation for 20 min, used the primary P2X4R antibody at 1:250 dilution, and used goat anti-rabbit secondary antibody at 1:700 dilution for P2X4R detection.
