# Supplementary material for: Characterization of the SIM-A9 cell line as a model of activated microglia in the context of neuropathic pain
Source: PLoS One. 2020 Apr 14;15(4):e0231597. doi: 10.1371/journal.pone.0231597 (PMC7156095; doi:10.1371/journal.pone.0231597)
Supplement: S6 Fig — A) Experiment scheme: SIM-A9 cells were cultured for 48 h and exposed to 2.5 to 25000 ng/mL LPS for 4 or 24 h. Cells were incubated with 25 nM to 250 μM ATP for 4 or 24 h. Cell Titer Glo ATP assay was performed immediately after exposure (B and E), 24 h (C and F), and 48 h post-ATP exposure (D and G). The viability of LPS/ATP treated cells was calculated relative to the control, untreated cells. Statistical analysis was performed using GraphPad Prism 8.1.2. Asterisks indicate significant differences (**** p<0.0001, *** p<0.001, ** p<0.005, * p<0.05) compared to the control. The data is representative of two independent experiments and is presented as mean ± standard deviation (SD) of at least n = 4 wells per group. (DOCX) [file pone.0231597.s006.docx]

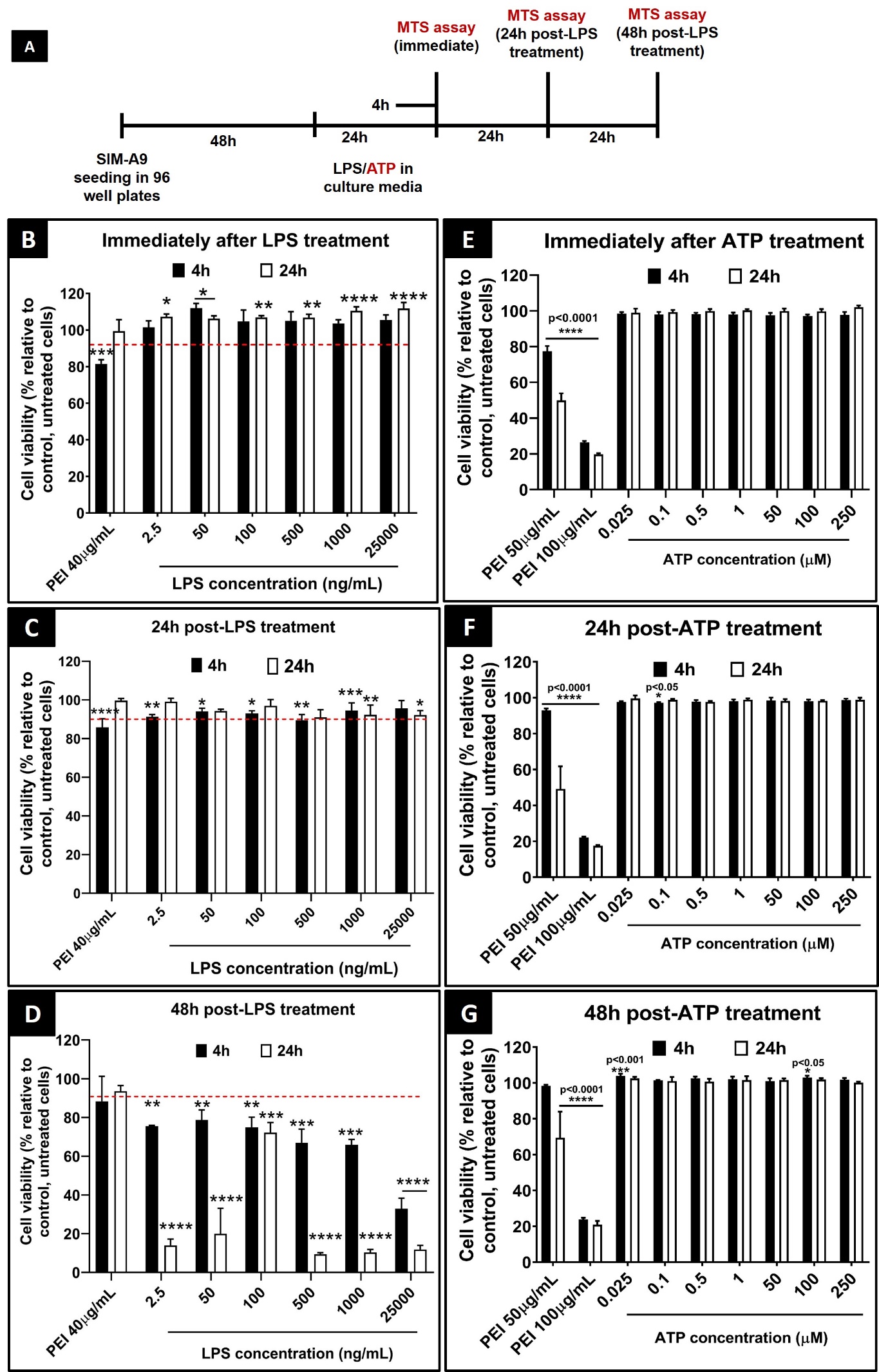


**S6 Fig.** **Cytocompatibility of LPS and ATP with SIM-A9 cells determined using MTS assay. A**) Experiment scheme: SIM-A9 cells were cultured for 48 h and exposed to 2.5 to 25000 ng/mL LPS for 4 or 24 h. Cells were incubated with 25 nM to 250 µM ATP for 4 or 24 h. Cell Titer Glo ATP assay was performed immediately after exposure (**B and E**), 24 h (**C and F**), and 48 h post-ATP exposure (**D and G**). The viability of LPS/ATP treated cells was calculated relative to the control, untreated cells. Statistical analysis was performed using GraphPad Prism 8.1.2. Asterisks indicate significant differences (**** p<0.0001, *** p<0.001, ** p<0.005, * p<0.05) compared to the control. The data is representative of two independent experiments and is presented as mean ± standard deviation (SD) of at least n=4 wells per group.
