# Supplementary material for: Characterization of the SIM-A9 cell line as a model of activated microglia in the context of neuropathic pain
Source: PLoS One. 2020 Apr 14;15(4):e0231597. doi: 10.1371/journal.pone.0231597 (PMC7156095; doi:10.1371/journal.pone.0231597)
Supplement: S7 Fig — Cells were incubated with LPS at different concentrations for 4 h in serum-containing treatment medium. After 48 h, the cells were dissociated from the plate and added into microcentrifuge tubes. Trypan blue dye was added at a 1:1 v/v ratio to the cell suspension and incubated for 5–10 min. Ten μL of the mixture was pipetted on a slide that was then were inserted in the Auto Cell counter (CountessII). (DOCX) [file pone.0231597.s007.docx]

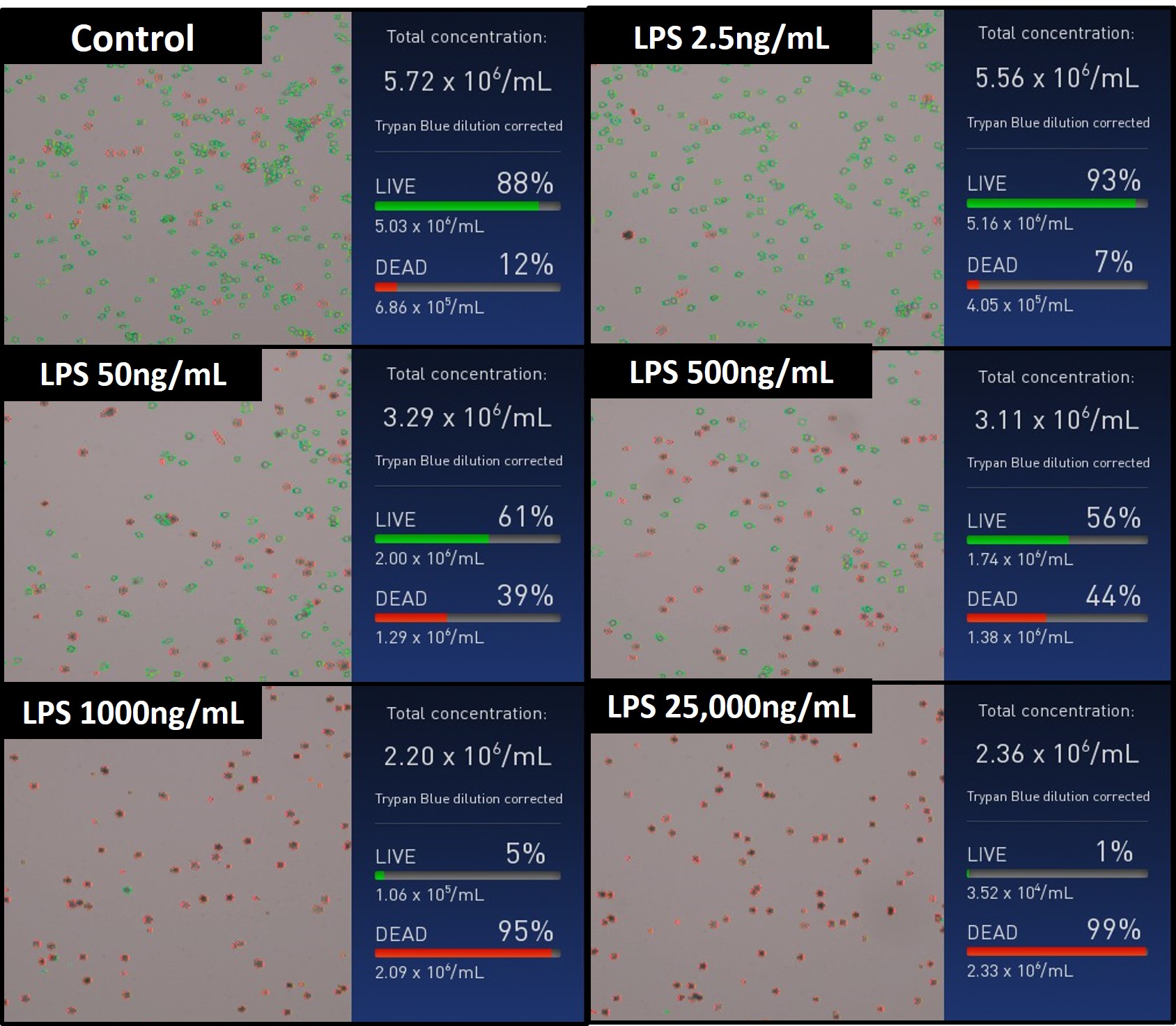


**S7 Fig**: **Effect of 4 h LPS exposure on SIM-A9 cells observed using Trypan blue assay.** LPS at a different concentration incubated for 4 h in serum-containing treatment medium. After 48 h, the cells were dissociated from the plate and added into microcentrifuge tubes. Trypan blue dye was added at a 1:1 ratio to the cell suspension and incubated for 5-10 min. Ten µL of the mixture was pipetted on a slide that was then were inserted in the Auto Cell counter (CountessII).
