# Supplementary material for: Characterization of the SIM-A9 cell line as a model of activated microglia in the context of neuropathic pain
Source: PLoS One. 2020 Apr 14;15(4):e0231597. doi: 10.1371/journal.pone.0231597 (PMC7156095; doi:10.1371/journal.pone.0231597)
Supplement: S8 Fig — Cells were incubated with LPS at different concentrations for 24 h in serum-containing treatment medium. After 48 h, the cells were dissociated from the plate and added into microcentrifuge tubes. Trypan blue dye was added at a 1:1 v/v ratio to the cell suspension and incubated for 5–10 min. Ten μL of the mixture was pipetted on a slide that was then were inserted in the Auto Cell counter (CountessII). (DOCX) [file pone.0231597.s008.docx]

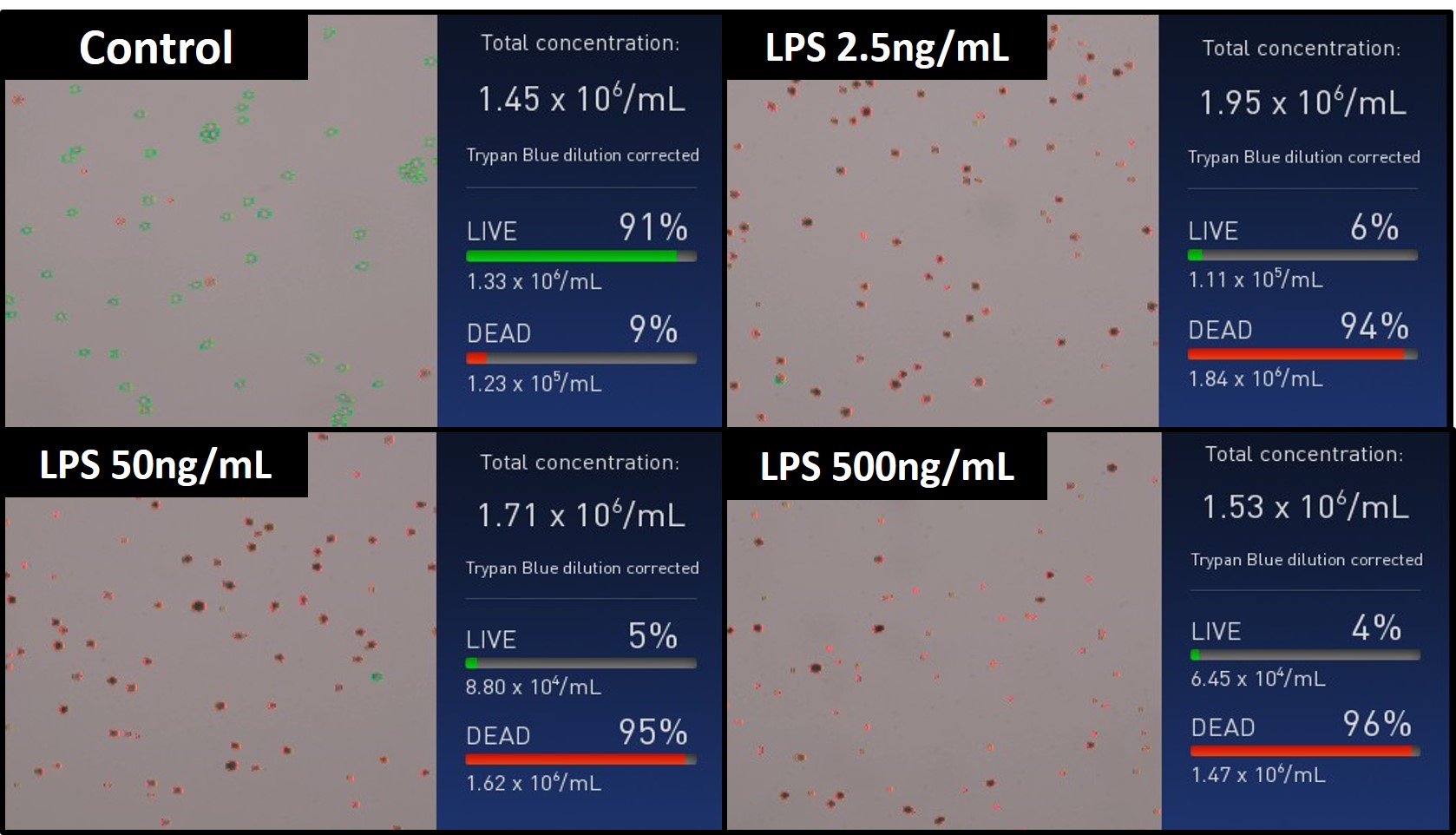


**S8 Fig**: **Effect of 24 h LPS exposure on SIM-A9 cells observed using Trypan blue assay.** LPS at a different concentration incubated for 24 h in serum-containing treatment medium. After 48 h, the cells were dissociated from the plate and added into microcentrifuge tubes. Trypan blue dye was added at a 1:1 ratio to the cell suspension and incubated for 5-10 min. Ten µL of the mixture was pipetted on a slide that was then were inserted in the Auto Cell counter (CountessII).

**Figure discussion:** We did not observe any visual reduction in cell numbers or morphological changes immediately and 24 h post-LPS treatments. **S7 and 8 Figs** show the images of Trypan blue-stained SIM-A9 cells treated with selected concentrations of LPS (2.5, 50, 500, 1000 and 25000 ng/mL). Countess II is an automated cell counter that operates on autofocusing-based quick and accurate cell counting and images trypan blue stained-cells. There was no change observed in the percentage (%) of dead cells between the control group and cells exposed to 2.5 ng/mL LPS. However, the %dead cells increased with an increase in LPS concentration. In **S8 Fig**, we noticed a complete loss in 2.5 ng/mL LPS-treated cells compared to the control. The complete loss in viability was also observed in cells treated at 50 and 50,000 ng/mL LPS concentrations.
