# Supplementary material for: Characterization of the SIM-A9 cell line as a model of activated microglia in the context of neuropathic pain
Source: PLoS One. 2020 Apr 14;15(4):e0231597. doi: 10.1371/journal.pone.0231597 (PMC7156095; doi:10.1371/journal.pone.0231597)
Supplement: S9 Fig — SIM-A9 cells were cultured for 48 h and exposed to 2.5 to 25,000 ng/mL LPS for 24 h. The treatment medium was replaced with the complete growth medium and incubated for 48 h. The images were acquired using an EVOS microscope at 10x magnification. The scale bar is 400 μm. (DOCX) [file pone.0231597.s009.docx]

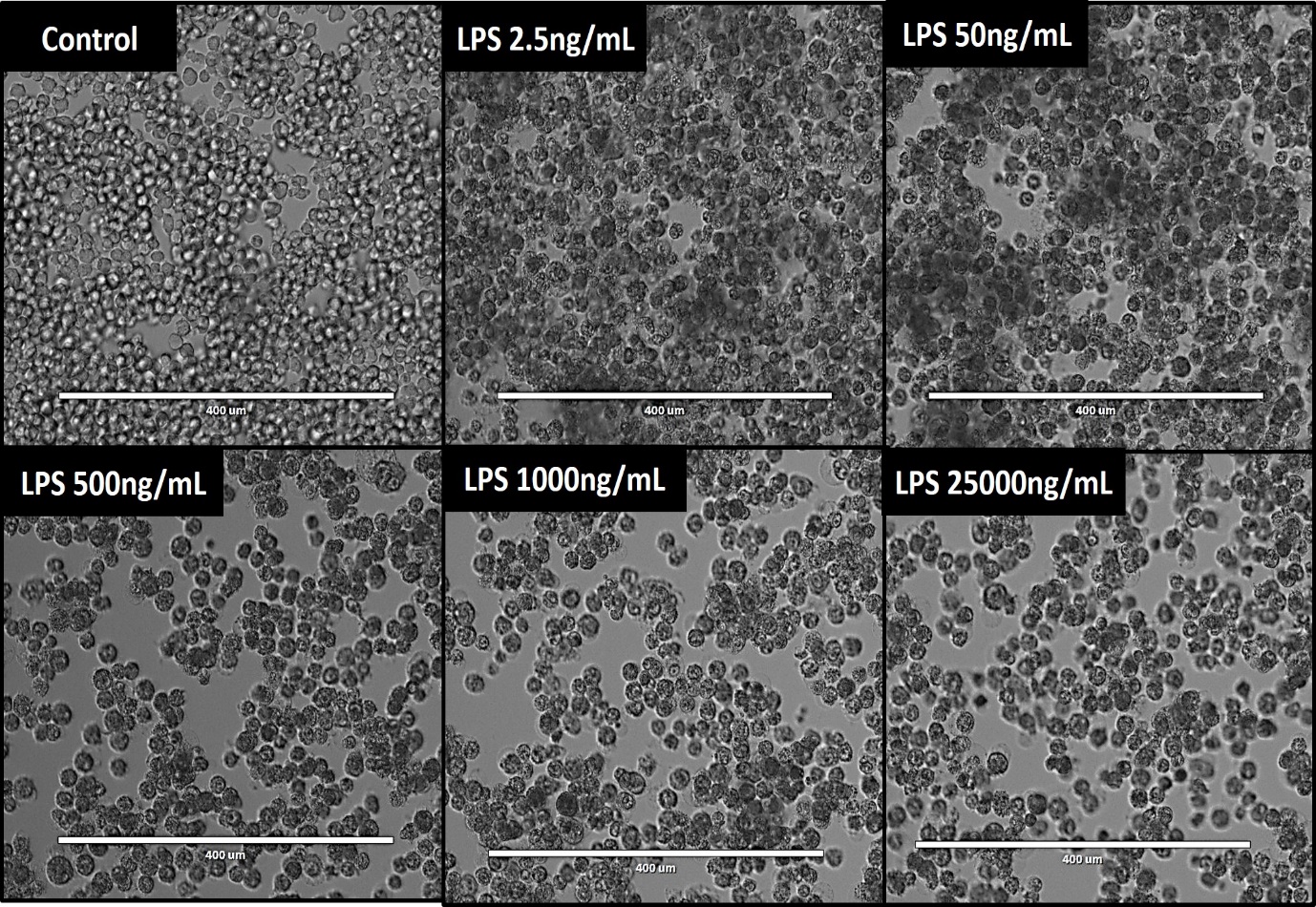


**S9 Fig**: **Microscopic images of control and LPS treated SIM-A9 in a 96-well plate.** SIM-A9 cells were cultured for 48 h and exposed to 2.5 to 25000 ng/mL LPS for 24 h. The treatment medium was replaced with the complete growth medium and incubated for 48 h. The images were acquired using an EVOS microscope at 10x magnification. The scale bar is 400µm.
