# Supplementary material for: Characterization of the SIM-A9 cell line as a model of activated microglia in the context of neuropathic pain
Source: PLoS One. 2020 Apr 14;15(4):e0231597. doi: 10.1371/journal.pone.0231597 (PMC7156095; doi:10.1371/journal.pone.0231597)
Supplement: S10 Fig — Cells were first exposed to LPS at the indicated concentrations for 24 h. The cells were then imaged 48 h-post exposure to LPS. The images were acquired using an EVOS microscope at 4x magnification. The scale bar is 1000 μm. (DOCX) [file pone.0231597.s010.docx]

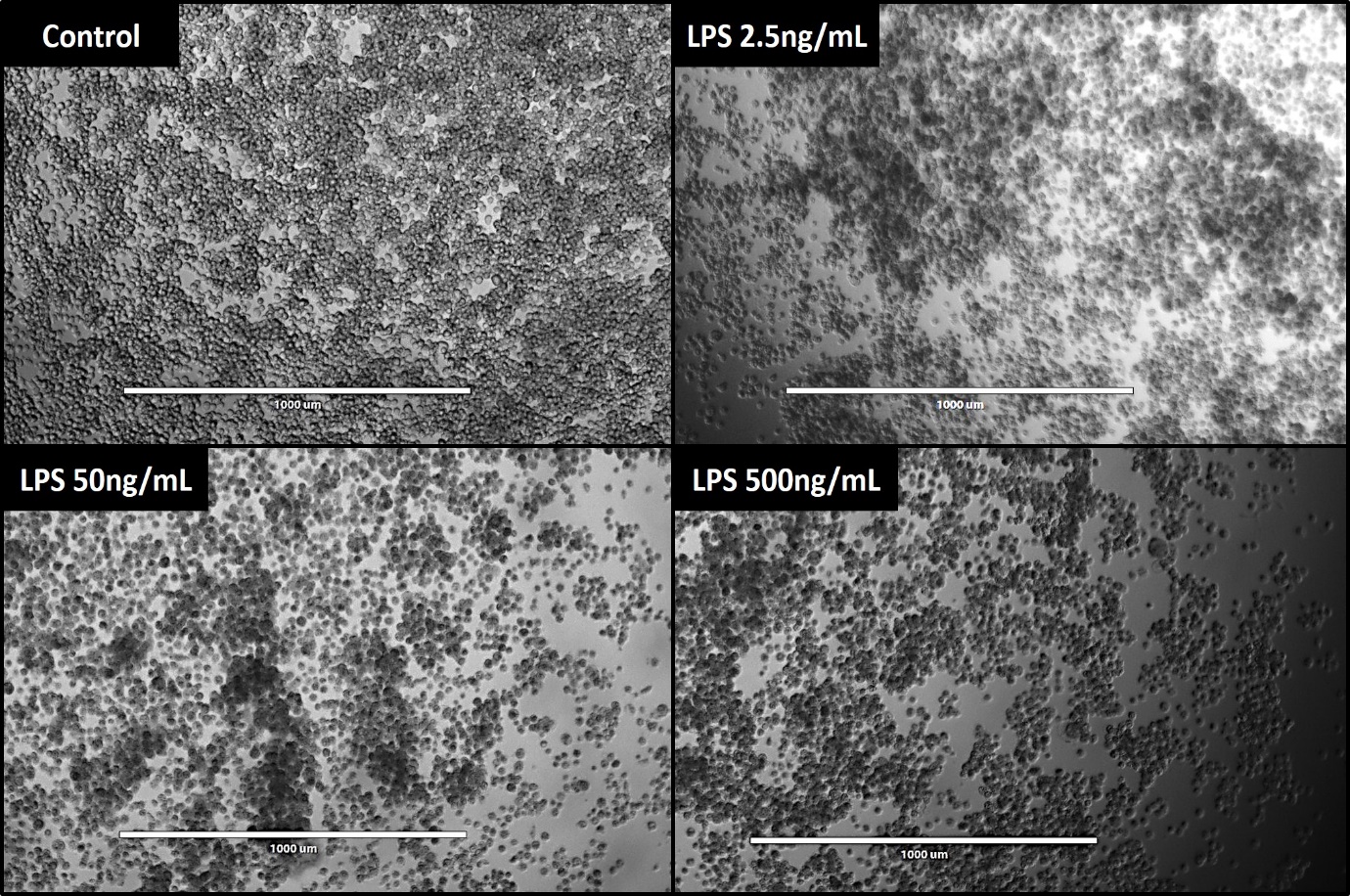


**S10 Fig**: **Microscopic images of control and LPS treated SIM-A9 cells in a 96-well plate.** Cells were first exposed to LPS at the indicated concentrations for 24 h. The cells were then imaged 48 h post-exposure to LPS. The images were acquired using an EVOS microscope at 4x magnification. The scale bar is 1000µm.

**Figure discussion**: **S9 and S10 Figs** depict a visual decrease in SIM-A9 cell number with an increase in LPS concentration compared to the control, untreated cells. Cells looked healthy, adhered fully and were homogeneously distributed in a control well at 48 h post-treatment. On the other hand, LPS-treated cells were semi-adhered, clustered and showed unequal-density throughout the well, reflecting cell stress and toxicity. In the 2.5 ng/mL LPS treated-well, we also observed cell loss near the circumference of the well. Cell numbers further decreased when treated with increasing concentrations of LPS ranging from 50 to 25,000 ng/mL.
