# Supplementary material for: Characterization of the SIM-A9 cell line as a model of activated microglia in the context of neuropathic pain
Source: PLoS One. 2020 Apr 14;15(4):e0231597. doi: 10.1371/journal.pone.0231597 (PMC7156095; doi:10.1371/journal.pone.0231597)
Supplement: S11 Fig — Effect of 50 μM ATP stimulation on SIM-A9 cells in serum-containing or serum-free treatment media determined using western blotting (A). Densitometry analysis of BDNF (B), BDNF dimer (C), and Iba1 (D) was performed using Image Studio 5.2. Normalized signal intensity in Y-axis represents the normalization of protein signal intensity to, first, α-tubulin signal intensity followed by normalization to the signal intensity of control, untreated cells. The order of loading the protein ladder and experimental samples were the same in the raw blot and S11A. (DOCX) [file pone.0231597.s011.docx]

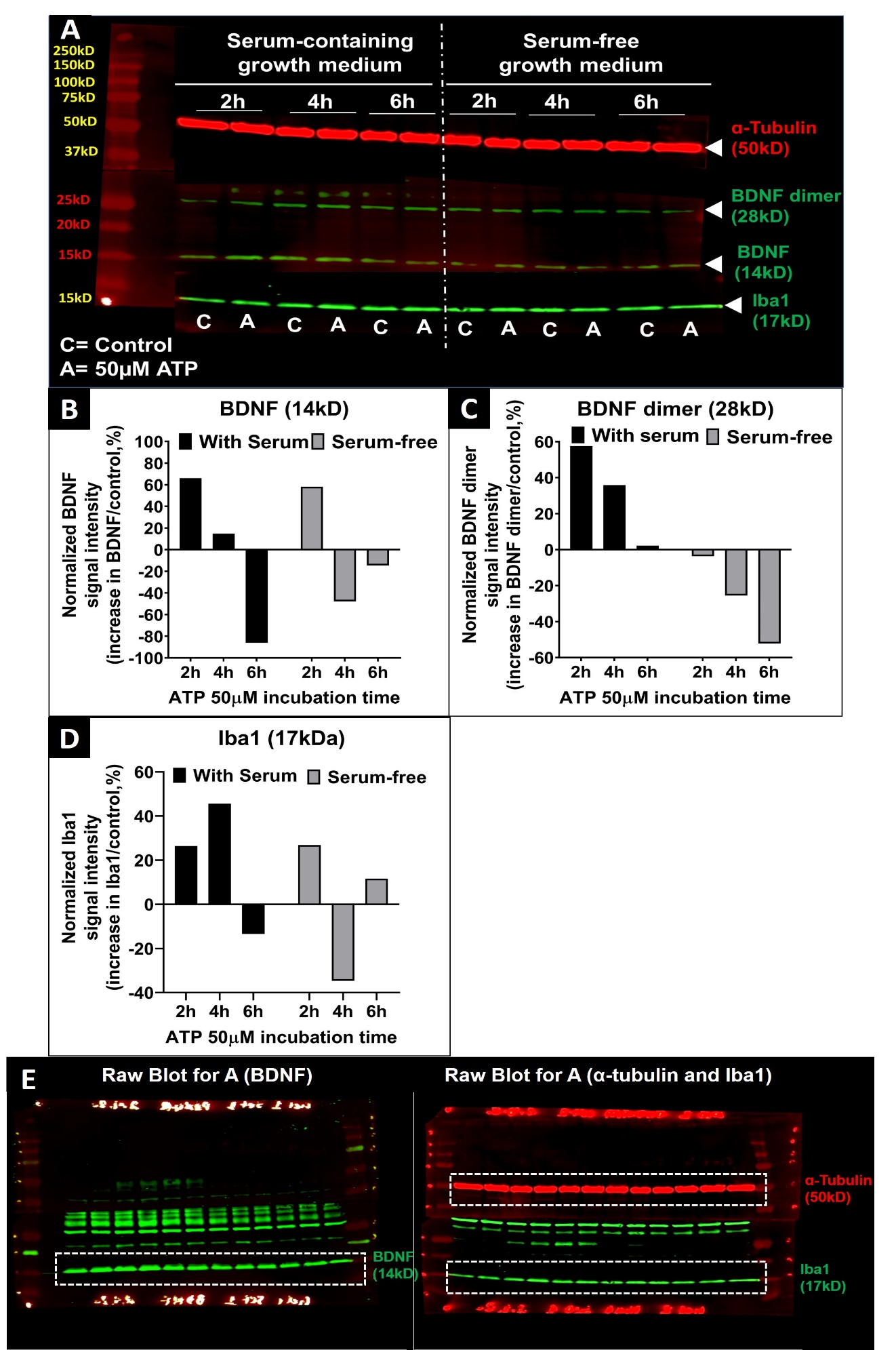


**S11 Fig:** **Effect of 50 µM ATP stimulation on SIM-A9 cells in serum-containing or serum-free treatment media determined using western blotting (A).** Densitometry analysis of BDNF (B), BDNF dimer (C), and Iba1 (D) was performed using Image Studio 5.2. Normalized signal intensity in Y-axis represents the normalization of protein signal intensity to, first, α-tubulin signal intensity followed by normalization to the signal intensity of control, untreated cells. (E) The raw blots for **S11A Fig** were shown as **Raw blot for A (BDNF)**, and **Raw blot for A (α-tubulin and Iba1)** respectively. The order and labeling of molecular weight markers in both the sides of **Raw blots A (BDNF) and B (α-tubulin and Iba1)** are the same as **S11A** **Fig.** The order of loading the protein ladder and experimental samples were the same in the raw blot and **S11A**.
