# Supplementary material for: Characterization of the SIM-A9 cell line as a model of activated microglia in the context of neuropathic pain
Source: PLoS One. 2020 Apr 14;15(4):e0231597. doi: 10.1371/journal.pone.0231597 (PMC7156095; doi:10.1371/journal.pone.0231597)
Supplement: S12 Fig — Normalized signal intensity in Y-axis represents a normalization of protein signal intensity to, first, α-tubulin signal intensity followed by normalization to the signal intensity of control/untreated cells at each time point. Data are presented as mean ± SD of n = 3 samples. (DOCX) [file pone.0231597.s012.docx]

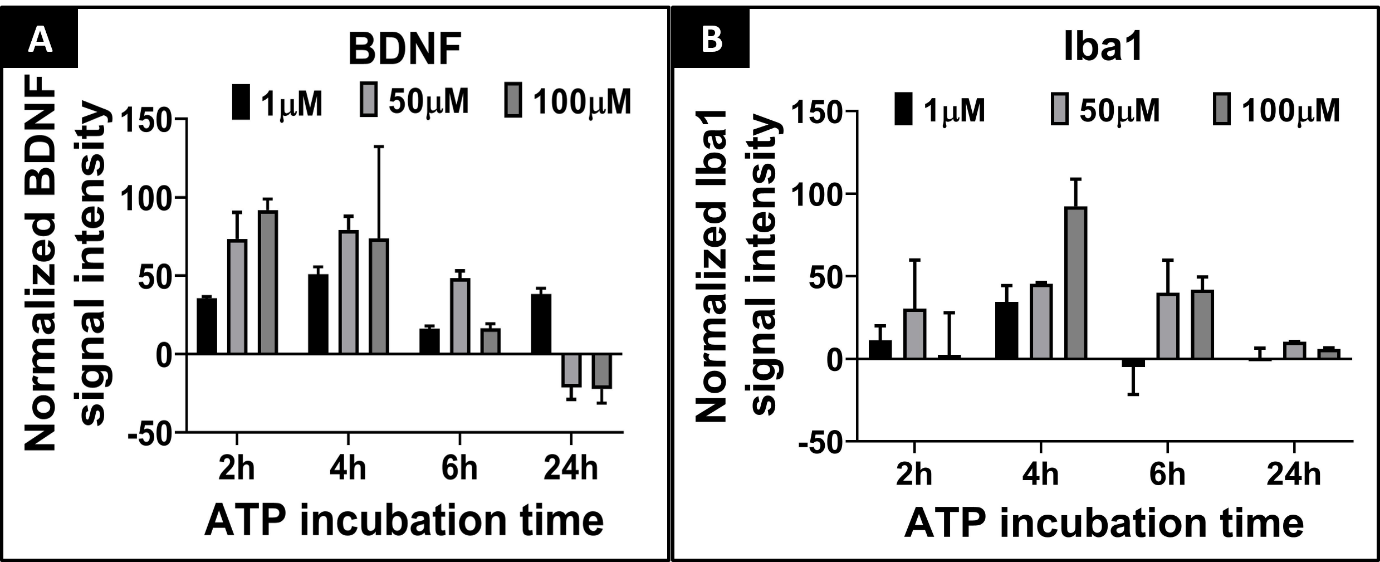


**S12 Fig.** **Densitometry analysis for BDNF and Iba1 of three independent western blot experiments demonstrating ATP-mediated modulation in expression levels of BDNF and Iba1.** Normalized signal intensity in Y-axis represents a normalization of protein signal intensity to, first, α-tubulin signal intensity followed by normalization to the signal intensity of control/untreated cells at each time point. Data are presented as mean ± SD of n=3 samples.
