# Supplementary material for: Characterization of the SIM-A9 cell line as a model of activated microglia in the context of neuropathic pain
Source: PLoS One. 2020 Apr 14;15(4):e0231597. doi: 10.1371/journal.pone.0231597 (PMC7156095; doi:10.1371/journal.pone.0231597)
Supplement: S13 Fig — The sign “X” indicates that the part of the blot was not depicted in Fig 5. (DOCX) [file pone.0231597.s013.docx]

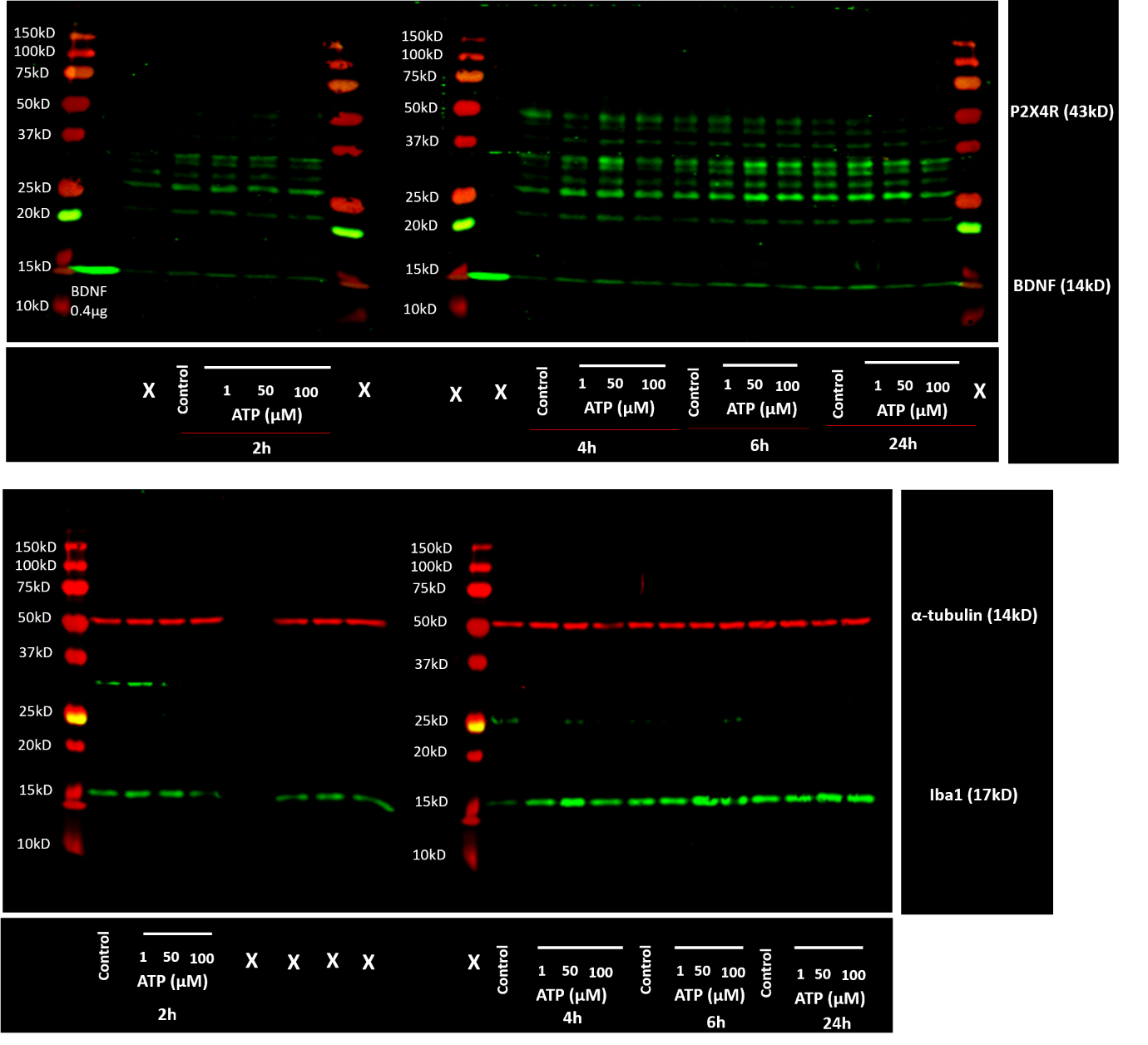


**S13 Fig.** **Raw western blots for Fig 5 in the main text.** The sign “X” indicates that the part of the blot was not depicted in **Fig 5**.
