# Supplementary material for: Characterization of the SIM-A9 cell line as a model of activated microglia in the context of neuropathic pain
Source: PLoS One. 2020 Apr 14;15(4):e0231597. doi: 10.1371/journal.pone.0231597 (PMC7156095; doi:10.1371/journal.pone.0231597)
Supplement: S14 Fig — The white squares in images were presented in Fig 6A in the main text demonstrating Iba1 expression, whereas red dotted squares in these images were depicted in Fig 6C in the main text demonstrating BDNF expression. (DOCX) [file pone.0231597.s014.docx]

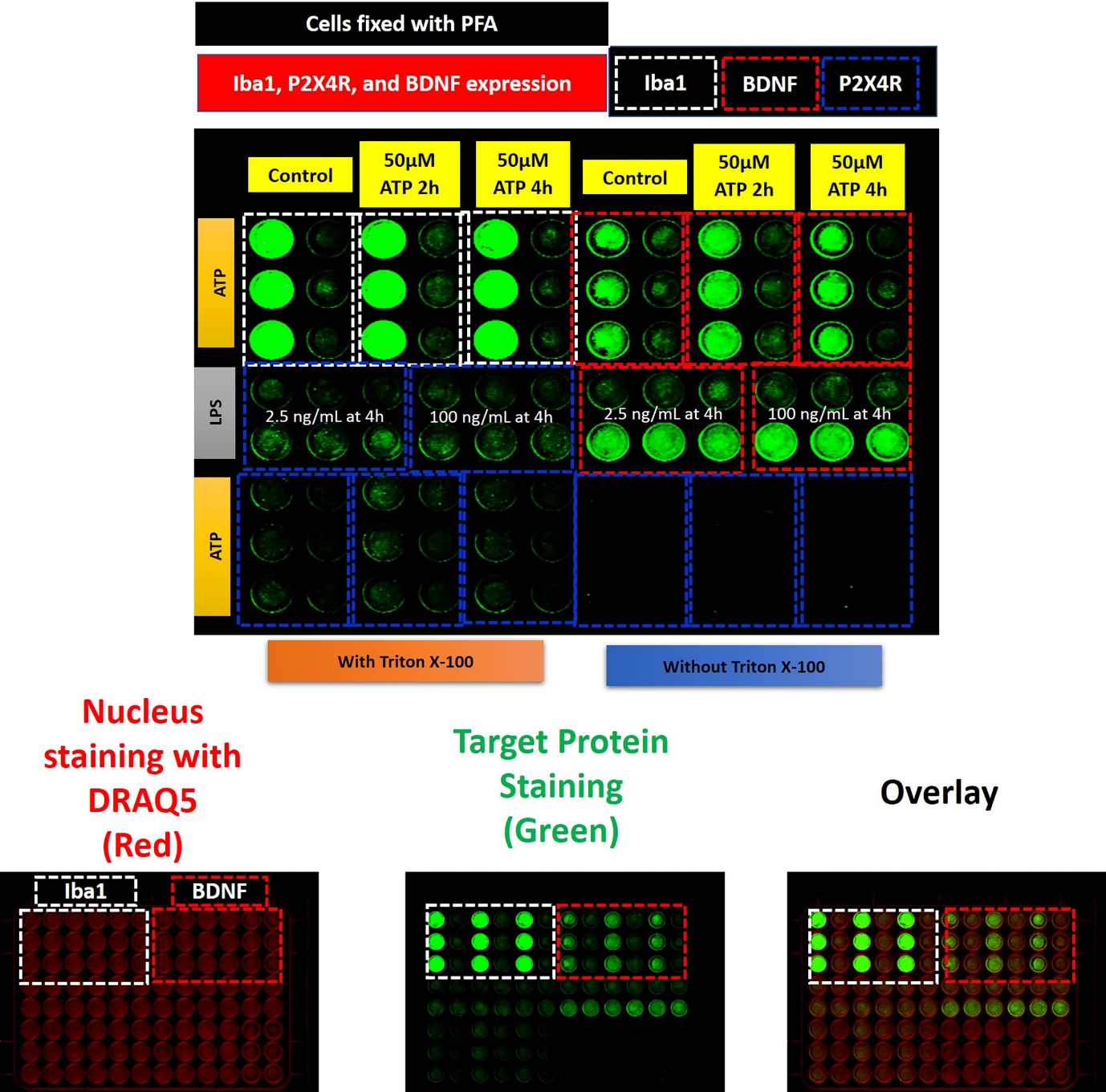


**S14 Fig.** **Raw images of ICW obtained using an Odyssey imager at 700 nm (red) and 800 nm (green) channels.** The white squares in images were presented in **Fig 6A in the main text** demonstrating Iba1 expression, whereas red dotted squares in these images were depicted in **Fig 6C in the main text** demonstrating BDNF expression.
