# Supplementary material for: Characterization of the SIM-A9 cell line as a model of activated microglia in the context of neuropathic pain
Source: PLoS One. 2020 Apr 14;15(4):e0231597. doi: 10.1371/journal.pone.0231597 (PMC7156095; doi:10.1371/journal.pone.0231597)
Supplement: S15 Fig — A) SIM-A9 cells were cultured for 48 h in a 96-well plate. The cells were treated with 2.5 and 100 ng/mL LPS for 4 h in complete growth media. Immediately after LPS treatment, cells were fixed, washed, blocked, and stained for cell nuclei (DAPI, red) and BDNF (green). B) Densitometry analysis was performed using Image Studio 5.2. Normalized BDNF signal intensity in the Y-axis represents the normalization of green signal intensity to DAPI signal intensity followed by subtraction of non-specific secondary staining. (DOCX) [file pone.0231597.s015.docx]

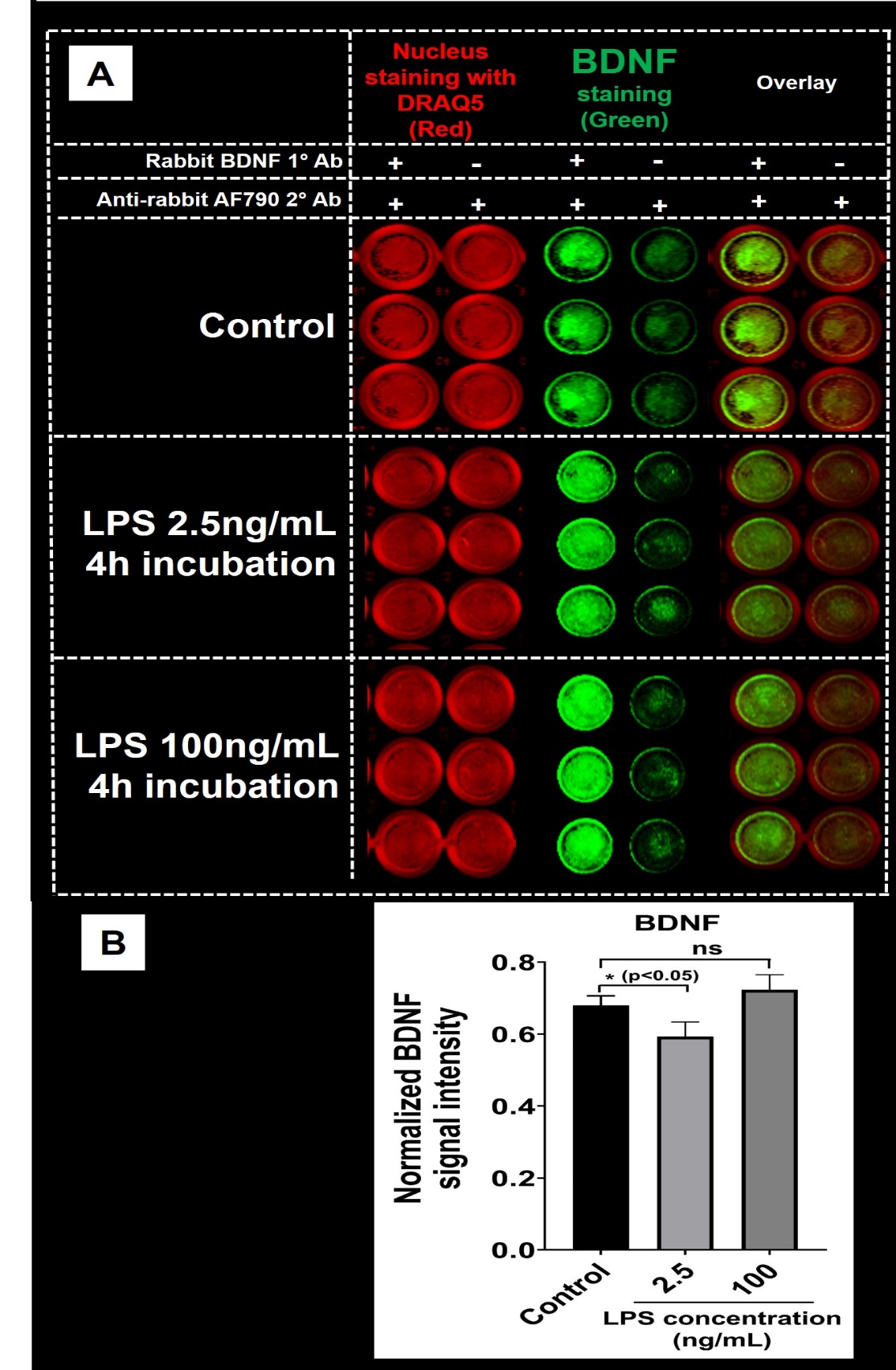


**S15 Fig.** **ICW of BDNF expression in 4h LPS treated SIM-A9 cells.** A) SIM-A9 cells were cultured for 48 h in a 96-well plate. The cells were treated with 2.5 and 100 ng/mL LPS for 4 h in complete growth media. Immediately after LPS treatment, cells were fixed, washed, blocked, and stained for cell nuclei (DAPI, red) and BDNF (green) proteins. B) Densitometry analysis was performed using Image Studio 5.2. Normalized BDNF signal intensity in the Y-axis represents the normalization of green signal intensity to DAPI signal intensity followed by subtraction of non-specific secondary staining.
