# Supplementary material for: Characterization of the SIM-A9 cell line as a model of activated microglia in the context of neuropathic pain
Source: PLoS One. 2020 Apr 14;15(4):e0231597. doi: 10.1371/journal.pone.0231597 (PMC7156095; doi:10.1371/journal.pone.0231597)
Supplement: S16 Fig — SIM-A9 cells were cultured for 48 h and exposed to 2.5 to 25000 ng/mL LPS for 4 h. MTS assay was performed 48 h post-LPS exposure. The viability of LPS-treated cells was calculated relative to the control group. Statistical analysis was performed using GraphPad Prism 8.1.2. Asterisks indicate significant differences (**** p<0.0001, *** p<0.001, ** p<0.005, * p<0.05) compared to the control. The data is representative of two independent experiments and is presented as mean ± standard deviation (SD) of at least n = 4 wells per group. (DOCX) [file pone.0231597.s016.docx]

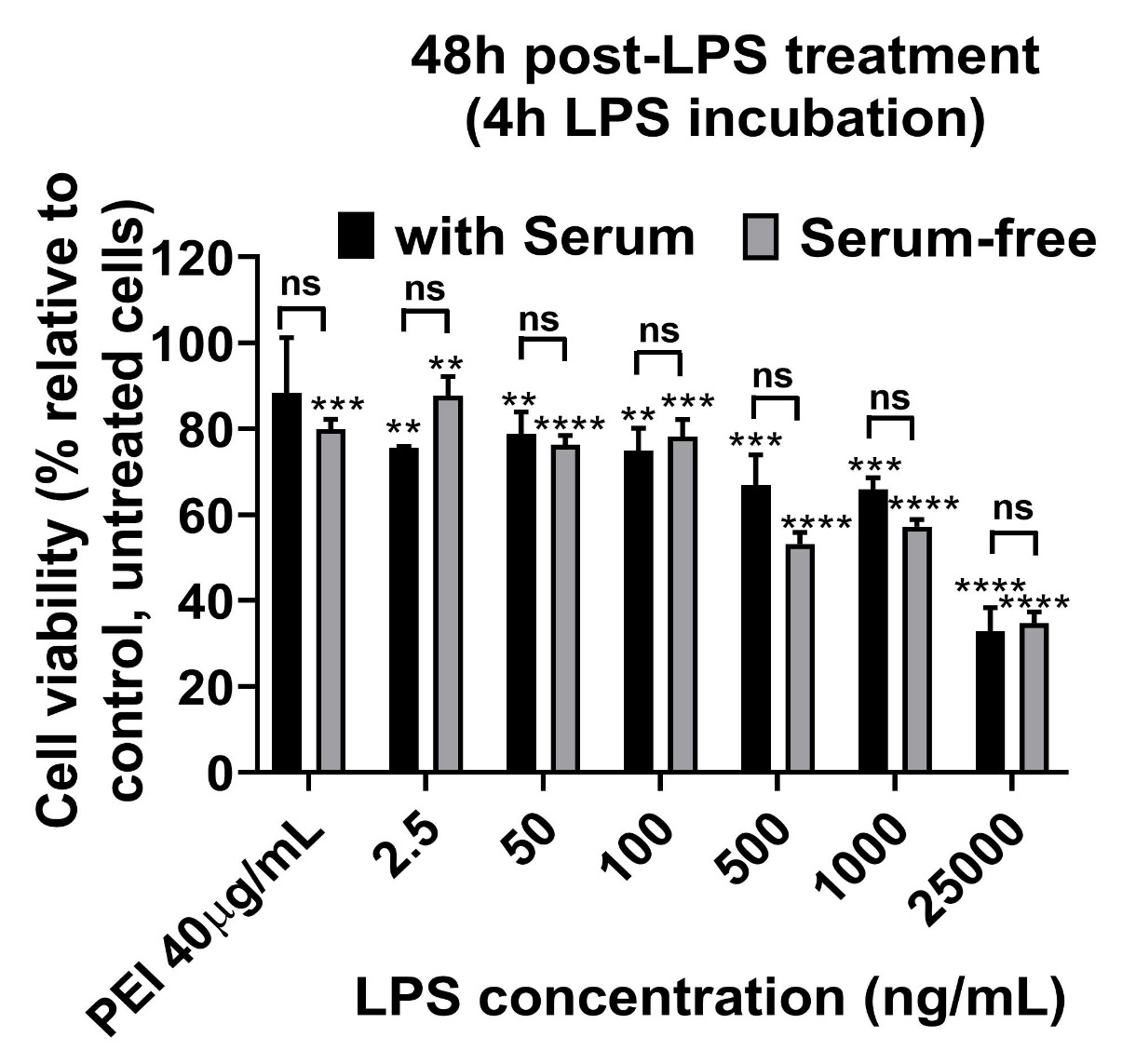


**S16 Fig**: **Cell viability of SIM-A9 48 h-post LPS treatment in serum-containing and serum-free medium determined using MTS assay.** SIM-A9 cells were cultured for 48 h and exposed to 2.5 to 25000 ng/mL LPS for 4 h. MTS assay was performed 48 h post-LPS exposure. The viability of LPS-treated cells was calculated relative to the control group. Statistical analysis was performed using GraphPad Prism 8.1.2. Asterisks indicate significant differences (**** p<0.0001, *** p<0.001, ** p<0.005, * p<0.05) compared to the control. The data is representative of two independent experiments and is presented as mean ± standard deviation (SD) of at least n=4 wells per group.
