# Supplementary material for: Characterization of the SIM-A9 cell line as a model of activated microglia in the context of neuropathic pain
Source: PLoS One. 2020 Apr 14;15(4):e0231597. doi: 10.1371/journal.pone.0231597 (PMC7156095; doi:10.1371/journal.pone.0231597)
Supplement: S1 Table — (DOCX) [file pone.0231597.s017.docx]

**S1 Table**: **Densitometry analysis of P2X4R band densities showing the effect of ATP stimulation at the 2 h time point.**

|  | **P2X4R signal intensity**  **(arbitrary values)** |
| --- | --- |
| **Control** | 8055 |
| **1 µM** | 6414 |
| **50 µM** | 12229 |
| **100 µM** | 9752 |
